# Comparative analysis of Panicum streak virus and Maize streak virus diversity, recombination patterns and phylogeography

**DOI:** 10.1186/1743-422X-6-194

**Published:** 2009-11-10

**Authors:** Arvind Varsani, Aderito L Monjane, Lara Donaldson, Sunday Oluwafemi, Innocent Zinga, Ephrem K Komba, Didier Plakoutene, Noella Mandakombo, Joseph Mboukoulida, Silla Semballa, Rob W Briddon, Peter G Markham, Jean-Michel Lett, Pierre Lefeuvre, Edward P Rybicki, Darren P Martin

**Affiliations:** 1 School of Biological Sciences, University of Canterbury, Private Bag 4800, Christchurch, New Zealand; 2 Electron Microscope Unit, University of Cape Town, Rondebosch, Cape Town, 7701, South Africa; 3 Department of Molecular and Cell Biology, University of Cape Town, Rondebosch, Cape Town, 7701, South Africa; 4 Department of Crop Production, Soil and Environmental Management, Bowen University, Iwo, Osun State, P.M.B. 284, Nigeria; 5 LASBAD Laboratory, Faculty of Sciences University of Bangui, BP 908 Bangui, Central African Republic; 6 National Institute for Biotechnology and Genetic Engineering, Jhang Road, P.O. Box 577, Faisalabad, Pakistan; 7 Department of Disease and Stress Biology, John Innes Centre, Norwich NR4 7UH, UK; 8 CIRAD, UMR 53 PVBMT CIRAD-Université de la Réunion, Pôle de Protection des Plantes, Ligne Paradis, 97410, Saint Pierre, La Réunion, France; 9 Institute of Infectious Disease and Molecular Medicine, University of Cape Town, Observatory, Cape Town, 7925, South Africa

## Abstract

**Background:**

*Panicum streak virus *(PanSV; Family *Geminiviridae*; Genus *Mastrevirus*) is a close relative of *Maize streak virus *(MSV), the most serious viral threat to maize production in Africa. PanSV and MSV have the same leafhopper vector species, largely overlapping natural host ranges and similar geographical distributions across Africa and its associated Indian Ocean Islands. Unlike MSV, however, PanSV has no known economic relevance.

**Results:**

Here we report on 16 new PanSV full genome sequences sampled throughout Africa and use these together with others in public databases to reveal that PanSV and MSV populations in general share very similar patterns of genetic exchange and geographically structured diversity. A potentially important difference between the species, however, is that the movement of MSV strains throughout Africa is apparently less constrained than that of PanSV strains. Interestingly the MSV-A strain which causes maize streak disease is apparently the most mobile of all the PanSV and MSV strains investigated.

**Conclusion:**

We therefore hypothesize that the generally increased mobility of MSV relative to other closely related species such as PanSV, may have been an important evolutionary step in the eventual emergence of MSV-A as a serious agricultural pathogen.

The GenBank accession numbers for the sequences reported in this paper are GQ415386-GQ415401

## Background

*Panicum streak virus *(PanSV) is one of seven known African streak virus species within the *Mastrevirus *genus of the *Geminiviridae*. The best studied and most economically relevant species amongst the African streak viruses is *Maize streak virus *(MSV) which seriously constrains maize production throughout most of sub-Saharan Africa [1]. Like MSV, African streak virus species such as *Panicum streak virus *(PanSV), *Sugarcane streak virus *(SSV), *Sugarcane streak Reunion virus *(SSRV) and *Sugarcane streak Egypt virus *(SSEV) are transmitted by various leafhopper species in the genus *Cicadulina *and have geographical ranges that are apparently restricted to Africa and its neighboring islands [1-7].

Whereas African streak virus species such as Eragrostis streak virus (ESV), Saccharum streak virus (SacSV), Urochloa streak virus (USV) and SSEV have been relatively poorly sampled and have therefore only ever been found in individual African countries [2,8-10], better sampling of MSV and PanSV has indicated that these species occur throughout sub-Saharan Africa [11,12]. PanSV and MSV display similar degrees of genetic diversity characterized by the existence of multiple discrete strains, many of which have distinctive geographical ranges [11,12]. Both species also have what appear to be largely overlapping host ranges. Unlike MSV, however, PanSV has no known economic relevance in that it has only ever been found in nature infecting wild grass species in the genera *Urochloa*, *Ehrharta *and *Panicum *[3,11,13].

Despite it not having any direct impact on African agriculture, the diversity and phylogeography of PanSV could still provide potentially useful information on other more economically important African streak viruses such as those that cause maize and sugarcane diseases. For example a recent comparative phylogeographic analysis of different MSV strains has indicated that the economically relevant maize adapted MSV-A strain is probably moving around Africa more freely than the closely related but *Digitaria *adapted MSV-B strain [12]. Comparative analyses of the diversity and phylogeography of different African streak virus species could therefore help identify the characteristics of MSV that facilitated its emergence as an important agricultural pathogen.

It has also been determined that African streak virus species such as PanSV have contributed indirectly to the evolution of MSV through genetic recombination [11,14]. Recombination is a major force in geminivirus evolution [15,16] and it appears to have played at least some role in the emergence of a number of serious geminiviral crop diseases [17-22]. At least seven of the eleven currently described MSV strains (including the important MSV-A strain) have apparently come into existence through recombination between two or more other strains [12]. It would be of great interest to determine whether such inter-strain recombination has featured as prominently in the diversification of other African streak viruses such as PanSV.

Here we use 23 full PanSV genome sequences sampled throughout Africa and one of its neighboring islands to show that there generally exist very similar patterns of diversity, recombination and geographical structure within PanSV and MSV populations. Our results indicate, however, that the maize adapted MSV-A strain is possibly unique amongst PanSV and MSV strains in both its total geographical range and the rates at which individual virus variants within the strain are moving across Africa.

## Results and discussion

### Discovery of five new PanSV strains

**Figure 1:**
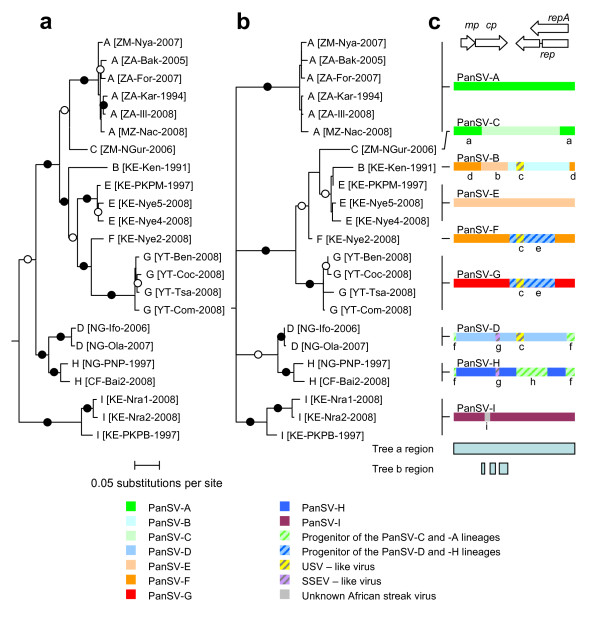
**Maximum likelihood phylogenetic trees (both constructed with the GTR+G_4 _nucleotide substitution model) indicating possible evolutionary relationships between 23 PanSV isolates**. (a) Tree constructed using complete genome sequences. Virus names take the form "Strain [country-region-year of isolation]" (b) A tree constructed using recombination-free portions of the genome indicated beneath the genome map and recombination mosaic cartoons in c. (c) Linearised genome cartoons depicting unique recombinant mosaics detected amongst the PanSV sequences. Colours represent as best as possible the origins of different genome regions. Letters below the depicted recombination events correspond to detailed descriptions of each of the events given in Additional file 7. For labels on the genome map: *mp *= movement protein gene, *cp *= coat protein gene, *rep *= replication associated protein gene, repA = RepA gene. Whereas branches marked with filled and open circles were supported in >90% and 70-89% of bootstrap replicates, respectively, branches with <50% bootstrap support have been collapsed. The tree was rooted on the Sugarcane streak Reunion virus isolate SSRV-Bas (not shown).

Sixteen full mastrevirus genome sequences were cloned and sequenced from *Brachiaria deflexa*, *Panicum maximum, Panicum trichocladium, Urochloa maxima *and *Ehrharta calycina *plants sampled from South Africa, Mozambique, Kenya, Nigeria, the Central African Republic and the Indian Ocean island of Mayotte (Table 1). All shared greater than 80% genome-wide identity with PanSV genomes currently deposited in public databases and were therefore all classified as being PanSV isolates. After confirming that plots of pairwise genetic similarity between all fully sequenced PanSV genomes closely matched those previously determined for MSV (Additional file 1), we used the 93% identity rule that has been used as a MSV strain demarcation criterion [14] to tentatively classify the PanSV isolates. This 93% identity threshold represents a logical, if not natural cutoff for classifying MSV and PanSV strains (Additional file 1) and it indicated that amongst the new sequences there potentially existed five new PanSV-strains (named PanSV-E to -I; Figure 1). It should be noted, however, that this classification scheme relied on the use of similarity measurements that exclude alignment gaps as missing data. Many other geminivirus classification schemes, such as the 75% and 89% thresholds endorsed by the ICTV for respectively demarcating mastrevirus and begomovirus species [23], do not specify how alignment gaps should be handled during similarity measurements. If we had included alignment gaps as a fifth character state - as is often done either accidentally or by design when arguments are made for or against new isolates being considered as new species - the 93% MSV/PanSV strain demarcation threshold would drop to between 90 and 91%.

**Table 1:** Accession numbers and sampling coordinates of various mastrevirus isolates used to detect recombination in PanSV.

Genebank accession #	Names/proposed names^*a*^	Host	Sampling coordinates	
			Lon	Lat
Y00514	MSV-A [ZA-SA-1986]	Maize	26.82875	-26.7688
EU628597	MSV-B [ZA-PlaB-g27-2006]	Unidentified	19.92809	-33.6634
AF007881	MSV-C [ZA-Set-1998]	*Setaria sp*	31.03495	-29.697
AF329889	MSV-D [ZA-Raw-1998]	Unidentified	19.74933	-33.7435
EU628626	MSV-E [ZA-MitA-g125-2006]	*Digitaria ciliaris*	31.00939	-29.8259
EU628629	MSV-F [NG-IntB-g88-2007]	*Urochloa maxima*	3.898865	7.406774
EU628631	MSV-G [TD-Mic24-1987]	*Digitaria sp*.	-7.8882	12.18787
EU628638	MSV-H [NG-Lag-g74-2007]	*Setaria barbata*	4.666667	8.916667
EU628639	MSV-I [ZA-NewA-g217-2007]	*Digitaria ciliaris*	30.89359	-29.8126
EU628641	MSV-J [ZW-Mic24-1987]	*Pennisetum sp*.	30.96333	-17.8761
EU628643	MSV-K [UG-BusD-2005]	*Eustachys petraea*	30.40586	0.458333
GQ415390	**PanSV-E [KE-Nye5-g359-2008]**	*Panicum maximum*	36.95382	-0.42397
GQ415393	**PanSV-G [YT-Tsa-g386-2008]**	*Panicum maximum*	12.83897	-45.1682
GQ415394	**PanSV-G [YT-Com-g383-2008]**	*Panicum maximum*	12.7824	-45.1298
GQ415395	**PanSV-G [YT-Coc-g385-2008]**	*Panicum maximum*	12.8343	-45.1399
GQ415396	**PanSV-G [YT-Ben-g384-2008]**	*Panicum maximum*	12.84907	-45.1907
GQ415392	**PanSV-F [KE-Nye2-g364-2008]**	*Panicum maximum*	36.94854	-0.41814
GQ415389	**PanSV-E [KE-Nye4-g363-2008]**	*Panicum maximum*	36.94688	-0.4004
GQ415399	**PanSV-I [KE-Nra1-g374-2008]**	*Brachiaria Deflexa*	37.04521	0.18032
GQ415399	**PanSV-I [KE-Nra2-g375-2008]**	*Brachiaria Deflexa*	37.04521	0.18032
GQ415391	**PanSV-E [KE-Jic10-PKPM-1997]^*b*^**	*Panicum maximum*	-	-
GQ415401	**PanSV-I [KE-Jic13-PKPB-1997]^*c*^**	*Panicum tricholadum*	-	-
GQ415397	**PanSV-H [CF-Bai2-Car11-2008]**	*Brachiaria Deflexa*	17.98736	3.848061
GQ415388	**PanSV-D [NG-Ola-g242-2007]**	*Urochloa maxima*	3.863739	7.411194
GQ415398	**PanSV-H [NG-Jic15-PNP-1997]^*d*^**	*Panicum maximum*	-	-
GQ415386	**PanSV-A [ZA-Ill-g263-2008]**	*Ehrharta calycina*	30.83108	-30.0653
GQ415387	**PanSV-A [MZ-Nac1-2009]**	*Panicum maximum*	-	-
EU224261	PanSV-A [ZA-Bak-M34-2005]	*Ehrharta calycina*	26.7522	-25.2321
EU224262	PanSV-A [ZA-For-g191-2007]	*Ehrharta calycina*	31.03868	-29.8545
EU224263	PanSV-A [ZM-Nya-g180-2007]	*Urochloa plantaginea*	32.8533	-18.5541
EU224264	PanSV-C [ZM-NGur-g169-2006]	*Urochloa plantaginea*	30.8402	-17.5216
EU224265	PanSV-D [NG-Ifo-g91-2006]	*Urochloa maxima*	5.776853	6.901831
L396381	PanSV-A [ZA-Kar-1994]	*Panicum maximum*	31.18425	-25.4951
X60168	PanSV-B [KE-Ken-1991]	*Panicum maximum*	-	-
EU244915	ESV [ZM-Gur-g186-2007]	*Eragrostis curvula*	31.1021	-17.8101
EU244913	SSRV-A [RE-Bas-R9-2006]	*Setaria barbata*	55.2715	-21.032
EU244916	SSRV-B [ZM-Nya-g177-2006]	*Paspalum conjugatum*	32.9715	-18.3213
AF072672	SSRV-A [RE-Reu]	Sugarcane	-	-
M82918	SSV-A [ZA-SN-1991]	Sugarcane	-	-
EU244914	SSV-B [RE-Pie-R5-2006]	*Cenchrus myosuroides*	55.4817	-21.3143
AF239159	SSEV [EG-Naga]	Sugarcane	-	-
AF037752	SSEV [EG-Giza]	Sugarcane	-	-
EU445697	USV [NG-Ipe-g226-2007]	*Urochloa deflexa*	4.45	7.51667
EU445699	USV [NG-Eji2-g248-2007]	*Urochloa deflexa*	4.32097	7.19889
EU445698	USV [NG-Eji-g230-2007]	*Urochloa deflexa*	4.32097	7.19889
EU445696	USV [NG-Ile-g240-2007]	*Urochloa deflexa*	4.24097	7.61211
EU445693	USV [NG-Iwo-g75-2006]	*Urochloa deflexa*	4.17803	7.62595
EU445692	USV [NG-Lag1-g74-2006]	*Urochloa deflexa*	4.66667	8.91667
EU445694	USV [NG-Lag2-g78-2006]	*Urochloa deflexa*	4.64886	8.92724
EU445695	USV [NG-Odo-g89-2006]	*Urochloa deflexa*	4.13646	7.46381

^*a*^Isolates sampled in the present study are indicated in bold type

^*b*^Isolate formerly named in P(K)P-M Pinner et al., (1988)

^*c*^Isolate formerly named P(K)P-B in Pinner et al., (1988)

^*d*^Isolate formerly named P(N)P in Pinner et al., (1988)

Although the newly described PanSV-I strain represented the most divergent group of PanSV isolates yet discovered, we found no major genomic features that could distinguish this or any of the other newly described PanSV strains from those already represented in public sequence databases (see additional files 2, 3, 4, 5 and 6 for annotated genome and probable expressed protein maps).

### Recombination between PanSV strains

It has been previously determined that recombination has featured prominently in the evolution of MSV strains [12] and that it may have also contributed substantially to the diversification of PanSV [11]. We therefore analysed the PanSV sequences for evidence of inter-species and inter-strain recombination events using a battery of recombination detection and analysis methods implemented in the program RDP3 [24]. We identified clear evidence of three inter-species (labeled a, b, d, e, f, and h in Figure 1 and Additional File 7) and six inter-strain recombination events (labeled a, b, d, e, f, and h in Figure 1) within the PanSV sequences.

The pattern of recombination we observed in PanSV is very similar to that which has been described for MSV [12]. For both species most detectable recombination events have involved intra-species sequence exchanges. The few inter-species recombination events that have been detected in both species have also all involved the exchange of small (<200 nt) tracts of sequence.

Another similarity between the two species is that many of the described strains have apparently arisen through inter-strain recombination events. For MSV all currently sampled isolates of seven of the eleven described strains (MSV-A, -F, -H, -J, K, C and D) share evidence of ancestral inter-strain recombination events that involved exchanges of genome fragments >30% of the full genome [12]. Likewise, exchanges of >30% genome size fragments are evident in all sampled representatives of five of the nine PanSV strains (PanSV-B, -C, -H, F and G).

The patterns of recombination seen in PanSV and MSV, where inter-species recombination events generally involve exchanges of only small genomic fragments (<10% of the full genome length), is quite different to that seen amongst related whitefly transmitted geminiviruses in the genus Begomovirus [15,16]. In these viruses inter-species recombination is very common and often involves exchanges of large (>30% of the full genome length) genome fragments. This difference is due, at least in part, to differences between the species classification criteria used for mastreviruses and begomoviruses. Whereas the main begomovirus species demarcation criterion is that DNA-A or DNA-A-like sequences (begomoviruses often have two component genomes where the DNA-A component of such genomes is largely homologous to mastrevirus genomes) sharing <89% identity belong to different species, the analogous mastrevirus species demarcation threshold is 75%. If the begomovirus classification scheme were applied to PanSV and MSV then, many of the inter-strain recombination events detectable in these species would be "upgraded" to inter-species recombination events.

It is still noteworthy, however, that detectable recombination events between more distantly related PanSV and MSV genomes have been less frequent and have tended to involve smaller sequence exchanges than recombination events between more closely related genomes. It is possible that the observed ratios of intra:inter species recombination events in PanSV and MSV might be partially attributable to mixed infections involving different mastrevirus species being rarer than mixed infections involving different strains of the same species. Although many of the different African streak virus species share hosts such as *Urochloa *and *Eragrostis *species, there are probably greater host-range differences between viruses in different species than there are between viruses within the same species. Such differences should surely influence the relative frequencies of mixed species and mixed strain infections and should therefore also influence the relative rates of inter-species and intra-species recombination events.

The most striking difference between the inter- and intra-species recombination events in these viruses is, however, not their relative frequencies, but rather the relative amounts of sequence that have been exchanged in these different recombination event categories. This pattern of recombination in fact conforms very well with the hypothesis that a major determinant of recombinant fitness is how well foreign DNA fragments function within the context of genomic backgrounds that they did not co-evolve within [25-29]. Functional nucleotide sequences tend to work best within genomes that are similar to the ones in which they evolved [25,27,30]. The probable reason for this is simply that the interaction networks that define the functionality of a particular nucleotide sequence within any given genomic context could potentially be disrupted if that sequence were placed into a genome where it was forced to interact with nucleotide sequences different from those it co-evolved with. As the relatedness between prospective parental sequences drops so too should the proportion of their genomes that could be exchanged without disrupting the delicate intra-genomic interactions required for optimal fitness [27,31]. The net effect of this process should be that amongst (presumably high fitness) genomes sampled from nature, one should tend to observe larger sequence exchanges between more closely related genomes than are detectable between less closely related ones. This is the exact pattern of recombination seen in both PanSV and MSV, suggesting that rather than inter-species recombination events being uncommon due entirely to different mastrevirus species only rarely infecting the same hosts, they are uncommon because of genetic constraints on the relative viability of inter-species recombinants.

### PanSV and MSV phylogenies display similar patterns of geographical structure

It has been previously demonstrated that there are strong signals of geographical structure within the phylogenetic trees of both the maize adapted MSV strain, MSV-A [14,22], and the grass adapted MSV strain, MSV-B [12]. These two strains differ, however, in the degree to which viruses have been moving across Africa [12]. Whereas no MSV-B isolates have ever been detected in West Africa, there have apparently been no movements of MSV-B isolates between East Africa, southern Africa and the Indian Ocean island of La Reunion since the initial spread of this strain to these three locations. Conversely, in the time since MSV-A first spread throughout the continent there have apparently been multiple instances where these viruses have moved between the major regions of Africa [12,22].

**Figure 2:**
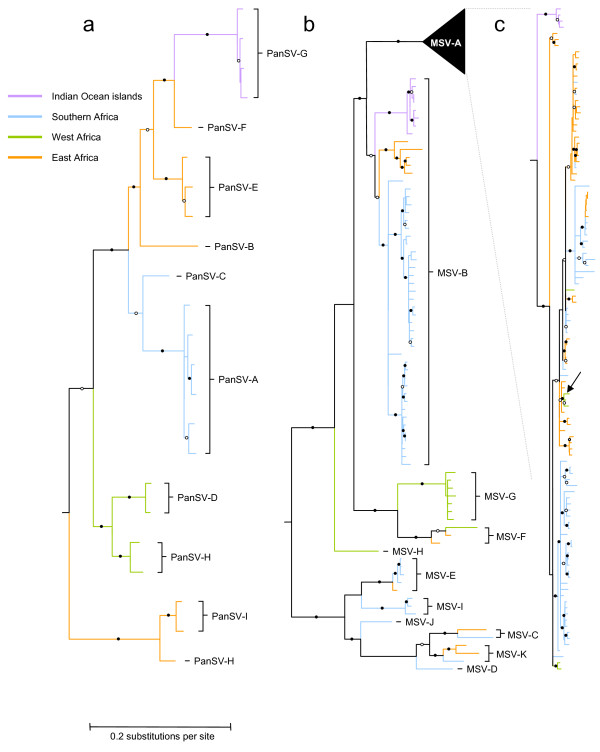
**Maximum likelihood phylogenetic trees (best fit nucleotide substitution models = GTR+G_4 _for PanSV tree and GTR+I+G_4 _for the MSV tree) depicting the sampling locations of 23 PanSV (tree a) and 181 MSV isolates (trees b and c)**. Branches are colored according to sampling locations (blue = southern African lineages, orange = East African lineages, green = West African lineages, purple = Indian Ocean island lineages). Wherever under a maximum parsimony criterion, it is <60% certain that ancestral sequences represented by tree nodes are from one of the four regions, the branches basal to that node have been left uncoloured. Whereas branches marked with filled and open circles were supported in >90% and 70-89% of bootstrap replicates, respectively, branches with <50% bootstrap support have been collapsed. The arrow on tree c indicates a clade of MSV-A sequences from West Africa nested within a clade of MSV-A sequences from East Africa indicating an instance of recent east to west movement of MSV-A isolates. All the trees were rooted on the Sugarcane streak Reunion virus isolate SSRV-Bas (not shown).

We sought to determine whether similar phylogeographic patterns exist amongst the currently sampled PanSV sequences. Taking note of the locations from which sequences were sampled, we compared the PanSV and MSV phylogenies (Figure 2) and noted some striking similarities between them with respect to the geographical ranges of the various distinct strain groupings represented.

Besides both MSV and PanSV strains clearly grouping according to their sampling locations, it is evident that PanSV (Figure 2a) and grass adapted MSV strains (Figure 2b) from East Africa, Southern Africa and the Indian Ocean islands are generally more closely related to one another than they are to viruses from West Africa. Taken together with the MSV data, the PanSV sample therefore provides additional evidence that, in general, African streak viruses may move more freely between East Africa, southern Africa and the Indian Ocean islands than they do between these regions and West Africa [12]. This pattern is notably different from that seen for both the maize adapted MSV-A strain and whitefly transmitted geminiviruses in the genus Begomovirus. Whereas island begomovirus populations display strong evidence of extensive isolation from mainland lineages [32,33], there is good phylogeographic evidence, particularly for cassava infecting begomoviruses, of lineages moving between the major regions of the continent [34]. Cassava infecting begomoviruses might, however, represent a special case in that cassava is propagated from cuttings and its viruses might therefore be moved more extensively by humans than viruses infecting seed propagated hosts.

Unlike with the wild-grass infecting MSV and PanSV strains, the maize adapted MSV-A strain (Figure 2c) has apparently moved quite extensively throughout the continent with the Indian Ocean islands being relatively more isolated than West Africa [12,35]. There is in fact clear evidence of at least one fairly recent movement of a MSV-A lineage from East Africa to West Africa (see arrow indicating the green clade nested within the orange clade in Figure 2c). Similar east to west movements across Africa have been detected in various other vector-born plant viruses including whitefly transmitted cassava infecting geminivirus species [34,36] and the beetle transmitted sobemovirus species, Rice yellow mottle virus [37,38]. It remains to be determined, however, whether movement of MSV-A and perhaps these other viruses too is natural or whether it is facilitated by human trafficking of infected plant material/viruliferous vectors [12,39]. It is also currently unknown whether PanSV, the grass adapted MSV strains and MSV-A are adapted to transmission by either different *Cicadulina *species or different biotypes within these species. MSV-A is transmitted with varying efficiencies by different *Cicadulina *species [40], and it remains a strong possibility that differences in the geographical distribution and migration routes of different preferred vector species might also account for differences in the movement patterns of these virus groups across East and West Africa.

The final subtle difference between the grass adapted MSV-strains and the PanSV dataset are the genetic distances between viruses found in different regions. Since the same demarcation threshold was used in both the MSV and PanSV strain classifications it is perhaps interesting that in no case was any PanSV strain detected in more than one of the surveyed regions. Members of each of the MSV-B -C, -E,-F and -K strains have been isolated across multiple geographical regions (three for MSV-B and two each for the rest). Assuming that the PanSV and grass adapted MSV strains are evolving at approximately the same rate, this observation indicates that over the time-scales represented by these phylogenies, MSV moves more frequently than PanSV between the regions examined. Without comparative analysis of PanSV and MSV substitution rates, however, it cannot be discounted that, rather than moving at different rates, the two species are simply evolving at different rates. It is also possible that with better sampling the isolates of different PanSV strains will, as is the case for MSV, be found in multiple different regions of Africa.

With these reservations noted, it is nevertheless interesting that just as MSV-A seems to be moving across Africa with less restraint than grass adapted MSV strains, the grass adapted MSV strains are in turn apparently moving more freely across the continent than PanSV strains. It is therefore possible that the evolution of epidemiological traits enabling MSV to move more rapidly than PanSV across Africa was important for the eventual evolution of still faster rates of MSV-A movement.

## Conclusion

Among 16 new PanSV isolates sampled across Africa and the Indian Ocean island of Mayotte we have potentially discovered five new PanSV strains. Together with other currently sampled PanSV genome sequences these new PanSV isolates indicate that there exist striking similarities between PanSV and MSV with respect to both detectable recombination patterns and degrees of geography-associated population structure. Although similarities between PanSV and MSV are perhaps unsurprising considering that these viruses share common leafhopper vector species and partially overlapping host-ranges, it remains interesting that both MSV strains in general, and the MSV-A strain in particular, seem to be less constrained in their movements across Africa than PanSV strains.

## Methods

### Virus Isolates

Sixteen grasses presenting with mild streak symptoms characteristic of African streak virus infections were sampled from various locations in Africa and the Indian ocean island of Mayotte: one *Brachiaria deflexa *sample from Central African Republic; four *Panicum maximum *samples from Mayotte; one *Urochloa maxima *and one *Panicum maximum *sample from Nigeria; four *Panicum maximum*, two *Brachiaria deflexa *and one *Panicum trichocladium *samples from Kenya; one *Ehrharta calycina *sample from South Africa and one *Panicum maximum *sample from Mozambique (locations and names are provided in Table 1). The Nigerian isolate, PanSV-H [NG-Jic15-PNP-1997], and the Kenyan isolates, PanSV-E [KE-Jic10-PKPM-1997] and PanSV-I [KE-Jic13-PKPB-1997] (respectively referred to as P(N)P, P(K)P-M and P(K)P-B in [5,41]), were sampled in ~1987 but were maintained for approximately ten years within *Panicum maximum *under glasshouse conditions at the John Innes Centre in Norwich prior to leaf tissues being harvested and frozen. Full length PanSV genomes were amplified from leaf tissues using rolling circle amplification, cloned and sequenced using methods described previously [42-44]. Briefly, total DNA was either extracted from leaf tissues using Extract-n-Amp™ Plant PCR Kit (Sigma-Aldrich Corporation, USA) or using a Qiagen Plant miniprep DNA kit (Qiagen, Germany) and circular DNA molecules were amplified using φ 29 DNA polymerase (TempliPhi™, GE Healthcare). The amplified concatamers were digested with *Bam*HI, *Sal*I or *Xho*I restriction enzymes to release ~2.7 kb PanSV genomes which were subsequently ligated to similarly linearised pGEM3 Zf(+) (from Promega Biotech). The cloned PanSV genomes were sequenced by Macrogen Inc (Korea) using primer walking. Sequences were assembled and edited using DNAMAN (version 5.2.9; Lynnon Biosoft) and MEGA (version 4)[45].

### Diversity Analysis

34 African streak virus full genome sequences, including all those available in GenBank for PanSV [3,11], and representative selections of MSV [12], USV [10], ESV [8], Sugarcane streak virus [4,8], Sugarcane streak Egypt virus [2], and Sugarcane streak Reunion virus [2,8], were obtained from GenBank. These were aligned together with the 16 new PanSV sequences using POA (vesion 2) [46] and edited by eye using MEGA. For purposes of assigning PanSV sequences to different strain groupings using the 93% rule of Martin *et al *[14], MEGA was also used to calculate the pair-wise differences between aligned PanSV genomes using p-distances with pair-wise deletion of gaps (as opposed to scoring gaps as a fifth nucleotide state). Alignments used in earlier phylogeographic analyses described in [12] and [22] were merged (with duplicate sequences being discarded) and realigned with MEGA.

### Recombination and phylogenetic analysis

Maximum likelihood phylogenetic trees were constructed using PHYML (version 1)[47] with automated best-fit model selection under the Akaike information criterion as described in [48].

Discreet recombination events were detected using the RDP [49], GENECONV [16], BOOTSCAN [50], MAXCHI [51], CHIMAERA [52], SISCAN [53], and 3SEQ [54] methods implemented in the program RDP3 (version 3.32; available from http://darwin.uvigo.es/rdp/rdp.html)[24]. Only potential recombination signals detected by at least three of the seven applied recombination detection methods, coupled with phylogenetic evidence of recombination were considered significant evidence of the signals representing genuine recombination events. Parental and recombinant sequences were identified from the sets of sequences used to detect recombination events as outlined in [55] and [56]. Recombination breakpoint positions and recombinant/parental designations were manually checked and adjusted where necessary using the extensive phylogenetic and recombination signal analysis features implemented in RDP3.

## Abbreviations

ESV: *Eragrostis streak virus*; *cp*: coat protein gene; LIR: long intergenic region; *mp*: movement protein gene; MSV: Maize streak virus; PanSV: *Panicum streak virus*; *rep*: replication associate protein gene; *repA*: RepA gene; SIR: short intergenic region; SSEV: *Sugarcane streak Egypt virus*; SSRV: *Sugarcane streak Reunion virus*; SSV: *Sugarcane streak virus*; USV: *Urochloa streak virus.sacSV*.

## Competing interests

The authors declare that they have no competing interests.

## Authors' contributions

AV, DPM, ALM, JML, SO, JZ, EKK, DPL, NM. JM, SS PGM and RWB collected isolates. AV, ALM, and LD cloned and sequenced the viruses. AV, PL and DPM conceived the project, AV and DPM analysed the data. AV and DPM prepared the manuscript and AV, PL, JML and DPM secured funding for the project's execution. EPR, PL and RWB provided ideas and comments during manuscript preparation. All authors other than PGM read and approved the final manuscript.

## Supplementary Material

Additional file 1 **Graph rationalizing the use with PanSV of the same 93% sequence identity strain demarcation threshold used for MSV**. Graph rationalizing the use with PanSV of the same 93% sequence identity strain demarcation threshold used for MSV. The red and blue splines respectively plots the frequencies of pairwise sequence identities shared amongst 23 PanSV (253 pairwise distances) and 99 MSV isolates (corresponding to the MSV dataset used in Varsani *et al *[2008] and accounting for 4851 pairwise distances) at a resolution of 1% identity. Identity values were calculated with pairwise exclusion of alignment gaps (as opposed to counting gaps as a fifth state as is often currently done, either by accident or design, by many geminivirologists). Click here for file

Additional file 2 **Full genome sequence alignments of 23 PanSV isolates**. Annotated full genome sequence alignments of 23 PanSV isolates. Sequences either known or believed to have some role in mastrevirus replication and transcription are marked together with a corresponding label on the nucleotide sequence alignments. To highlight differences between the sequences, wherever nucleotides in a particular alignment column are identical to that of PanSV-A [ZM-Nya-g180-2007] they are replaced with a "-" character. In columns where they differ from PanSV-A [ZM-Nya-g180-2007] they are shown in lower case. "." characters indicate where gaps were inserted to align the sequences. [1] Stenger, *et al*., 1991. Proc. Natl. Acad. Sci. USA 88:8029; [2] Sunter, *et al*. 1985. Nucl. Acids Res. 13:4645; [3] Argüello-Astorga et al. 1994. Virology 203:90; [4] Suárez-López *et al*. 1995. Virology. 208:303; [5] Morris-Krsinich *et al*. 1984. Nucleic Acids Res. 13:7237; [6] Boulton *et al*. 1989. J. Gen. Virol. 70:2309; [7] Wright *et al*.1997 Plant J. 12:1285; [8] Donson *et al*. 1984. EMBO J. 3:3069; [9] Dekker *et al*.1991. Nucl. Acids Res. 19:4075; [10] Fenoll *et al*. 1990. Plant. Mol. Biol. 15:865. Click here for file

Additional file 3 **Annotated predicted movement protein amino acid sequence alignments**. Annotated predicted movement protein amino acid sequence alignments of 23 PanSV isolates. The hydrophobic, potentially membrane spanning internal domain of the sequences is highlighted. [1] Wright et al.1997. Plant J. 12:1285. Click here for file

Additional file 4 **Annotated predicted coat protein amino acid sequence alignments**. Annotated predicted coat protein amino acid sequence alignments of 23 PanSV isolates. The potential nuclear localization signal and DNA binding domains (inferred by analogy with those determined for MSV) are highlighted on the sequence. [1] Liu et al. 1999. Mol. Plant Microbe Interact. 12:894; [2] Liu et al. 1997. J. Gen. Virol. 78:1265. Click here for file

Additional file 5 **Annotated predicted replication-associated protein amino acid sequence alignments**. Annotated predicted replication-associated protein amino acid sequence alignments of 23 PanSV isolates. Potential rolling-circle replication motifs and interaction domains inferred by analogy with MSV and Wheat dwarf virus are highlighted. [1] Koonin & Ilyina. 1992. J Gen Virol, 73:2763; [2] Horvath et al. 1998. Plant Mol. Biol. 38:699; [3] Xie et al. 1995. EMBO J. 14:4073; [4] Gorbalenya & Koonin. 1989. Nucl. Acids Res. 17:8413. Click here for file

Additional file 6 **Annotated predicted RepA amino acid sequence alignments**. Annotated predicted RepA amino acid sequence alignments of 23 PanSV isolates. Potential rolling circle replication motifs and interaction domains inferred by analogy with MSV and Wheat dwarf virus are highlighted. [1] Koonin & Ilyina. 1992. J Gen Virol, 73:2763; [2] Horvath et al. 1998. Plant Mol. Biol. 38:699; [3] Xie et al. 1995. EMBO J. 14:4073; [4] Xie et al. 1999. Plant Mol. Biol. 39:647. Click here for file

Additional file 7 **Recombination in PanSV**. Details of the PanSV recombination events detected in this study including approximate breakpoint positions, parental-like sequences, and p-values for various recombination detection tests. Click here for file
