# Supplementary material for: Comparative analysis of Panicum streak virus and Maize streak virus diversity, recombination patterns and phylogeography
Source: Virol J. 2009 Nov 10;6:194. doi: 10.1186/1743-422X-6-194 (PMC2777162; doi:10.1186/1743-422X-6-194)
Supplement: Additional file 1 — Graph rationalizing the use with PanSV of the same 93% sequence identity strain demarcation threshold used for MSV. Graph rationalizing the use with PanSV of the same 93% sequence identity strain demarcation threshold used for MSV. The red and blue splines respectively plots the frequencies of pairwise sequence identities shared amongst 23 PanSV (253 pairwise distances) and 99 MSV isolates (corresponding to the MSV dataset used in Varsani et al [2008] and accounting for 4851 pairwise distances) at a resolution of 1% identity. Identity values were calculated with pairwise exclusion of alignment gaps (as opposed to counting gaps as a fifth state as is often currently done, either by accident or design, by many geminivirologists). [file 1743-422X-6-194-S1.ppt]

## Slide 1
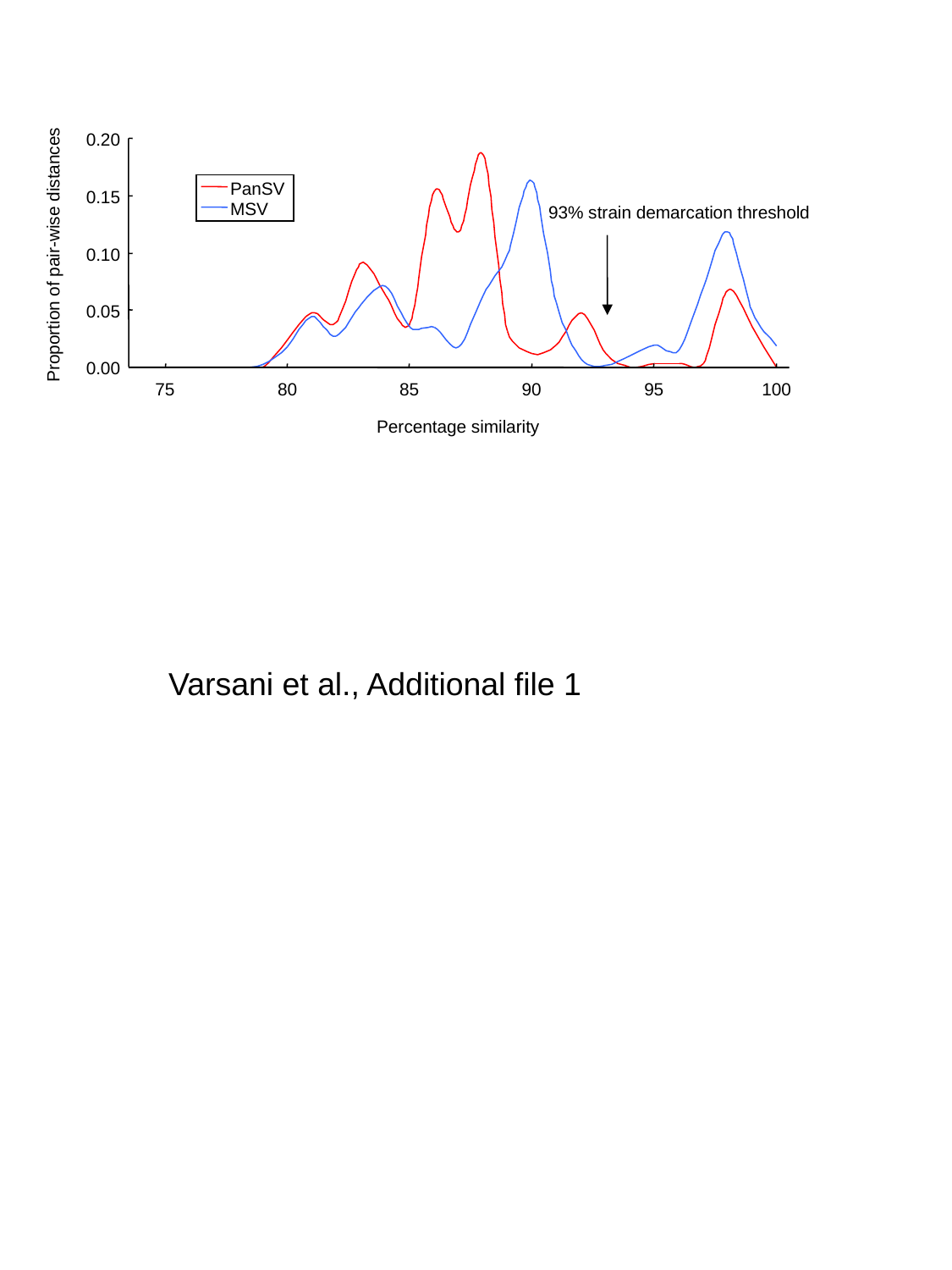

0.20
PanSV
MSV
0.15
93% strain demarcation threshold
Proportion of pair-wise distances
0.10
0.05
0.00
75
80
85
90
95
100
Percentage similarity
Varsani et al., Additional file 1
