# Supplementary material for: Comparative analysis of Panicum streak virus and Maize streak virus diversity, recombination patterns and phylogeography
Source: Virol J. 2009 Nov 10;6:194. doi: 10.1186/1743-422X-6-194 (PMC2777162; doi:10.1186/1743-422X-6-194)
Supplement: Additional file 2 — Full genome sequence alignments of 23 PanSV isolates. Annotated full genome sequence alignments of 23 PanSV isolates. Sequences either known or believed to have some role in mastrevirus replication and transcription are marked together with a corresponding label on the nucleotide sequence alignments. To highlight differences between the sequences, wherever nucleotides in a particular alignment column are identical to that of PanSV-A [ZM-Nya-g180-2007] they are replaced with a "-" character. In columns where they differ from PanSV-A [ZM-Nya-g180-2007] they are shown in lower case. "." characters indicate where gaps were inserted to align the sequences. [1] Stenger, et al., 1991. Proc. Natl. Acad. Sci. USA 88:8029; [2] Sunter, et al. 1985. Nucl. Acids Res. 13:4645; [3] Argüello-Astorga et al. 1994. Virology 203:90; [4] Suárez-López et al. 1995. Virology. 208:303; [5] Morris-Krsinich et al. 1984. Nucleic Acids Res. 13:7237; [6] Boulton et al. 1989. J. Gen. Virol. 70:2309; [7] Wright et al.1997 Plant J. 12:1285; [8] Donson et al. 1984. EMBO J. 3:3069; [9] Dekker et al.1991. Nucl. Acids Res. 19:4075; [10] Fenoll et al. 1990. Plant. Mol. Biol. 15:865. [file 1743-422X-6-194-S2.doc]

A (G180) ACC-GCTCACACCCCATGCGAGCCGCGGCTCTGTGCGGCGAGCGGTCCCCGGATGTTCTTACCCCGGTGCGATTTC-TTT 80


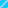


Virion strand origin of replication[1]

Inverted repeat[2]

Iterons[3]

GC-rich sequence sequence

T-Tracts[4]

A (M34) -------------------------------------------------------------------------------- 80


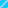


A (G191) -------------------------------------------------------------t------------------ 80

A (KAR) ------------------------a----c-----t-------------------------------------------- 80

A (G263) ------------------------a----c-----t-------------------------------------------- 80

A (NAC1) -------------------------------------------------------------------------------- 80

C (G169) ------------------------a----c-----t-------------------------------------------- 80

B (KEN) ---------------------------------------------------------t-ac------------------- 80

E (JIC10) ---------------------------------------------------------t-ac------------------- 80

E (G359) ------------------------------------------------t----------ac------------------- 80

E (G363) ---------------------------------------------------------t-ac------------------- 80

F (G364) -----------------------------c-----------------------------ac------------------- 80

G (G384) ---c-----------------------------------------------t---g--c----------t---------c 80

G (G385) ---c-----------------------------------------------t---g--c----------t---------c 80

G (G386) ---c-----------------------------------------------t---g--c----------t----c----c 80

G (G383) ---c-----------------------------------------------t---g--c----------t---------c 80

D (G91) ------------------------a----c-----t------------tgctc--ccaggctg----------------- 80

D (G242) ------------------------a----c-----t------------tacac--c-t-gt-t----------------- 80

H (JIC15) ------------------------a----------t------------tactc--c-t-gc-t----------------- 80

H (CAR11) ------------------------a----c-----t------------tacac--c-t-gt-t----------------- 80

I (G374) --a---------t---------------tc--a---cc----------t--tt--c-t-gtt-ta-----------c--- 80

I (G375) --a---------t---------------tc--a---cc----------t--tt--c-t-gtt-ta--------------- 80

I (JIC13) --a---------t----------------c-aa---cc-------------tt--c-t-gtt-ta-----------c--- 80

TATA box[5,7] sequence

Movement protein start codon[6]

A (G180) AGCTGCGTCTTCTTTAGCAGCTGC-CCCCACTGTCCTATAAGTTGCTCCCCCG-GTGCGATTCCGCATCATGGATGCTAG 160

A (M34) -------------------------------------------------------------------------------- 160

A (G191) ---------g---------------------------------------------------------------------- 160

A (KAR) --------g----------------------------------------------------------------------- 160

A (G263) -----------------------------------------------t-------------------------------- 160

A (NAC1) -------------------------------c----------------------------------------t------- 160

C (G169) -------------------------------------------------------------------------------- 160

B (KEN) --------g---------------------------------a-----t------g------------------------ 160

E (JIC10) --------g---------------------------------a-------t----------------------------- 160

E (G359) --------g---------------------------------a-------t----------------------------- 160

E (G363) --------g------------------a--------------a-------t----------------------------- 160

F (G364) --------g-----------------tg-c------------a-----tg--a--------------------------- 160

G (G384) g------ggac----t-----------------------------tg---t---------------------------ca 160

G (G385) g------agcc----t-----------------------------tg---t---------------------------ca 160

G (G386) g------ggac----t-----------------------------tg---t---------------------------ca 160

G (G383) g------ggac----t----t-----------a------------tg---t---------------------------ca 160

D (G91) -------c-g-----------------a--------------------t--g-a--------g-----------g----- 160

D (G242) -------c-g--------------------------------a-----t--g-a--------g-----------g----- 160

H (JIC15) -------ag---------------------------------a--tc-t--g-a---------t----------g----- 160

H (CAR11) -------ag---------------------------------a--------g-a---------t----------g----c 160

I (G374) --------g------------c--a-t--tt-----------a------t-g-a---------ta---------g--c-- 160

I (G375) --a-----g------------c--a-t--tt-----------a------t-g-a--------gta---------g--c-- 160

I (JIC13) --------g------------c--ggt---t-----------a----t-t-g-a---------ta---------g--c-- 160

A (G180) CAGCACCACTCCT---TTTCCTTTCCCTCAGCCGCCTCGGGTACCCTCTGCAGCTCCGGTCGCCGGAGGCTTGCCGTGGA 240

A (M34) ----t--------------------------------------------------------------------------- 240

A (G191) ----------------------------------------------------------------------c--------- 240

A (KAR) --------------------------------------------------------------------cgc--------- 240

A (G263) ----------------------------------------------------------------------c--------- 240

A (NAC1) ----------------------------------------------------------------------c--------- 240

C (G169) ----------------------------------------------------------tc----------c--------- 240

B (KEN) ----cagtact--gct--g----at--------c--g---------------------tc-------c-gc--------- 240

E (JIC10) --a-cagtact--gct--g--c-at--------a--c---------------------tc---------tc--------- 240

E (G359) ----cagtact--gct--g--g-at--------a--c---------------------tc---------tc--------- 240

E (G363) ---tcagtact--gct--a--g-at--------a--c---------------------tc---------tc--------- 240

F (G364) ----cagtact--gct--g--c-at--------c--g---------------------tc----------c--------- 240

G (G384) a----g-tact-atct--g--c-at--gtctg-c--g---------------------tc-------------------- 240

G (G385) a----g-tact-atct-----c-at--gtctg-c--g---------------------tc-------------------- 240

G (G386) a----g-tact-atct--g--c-at--gtctg-c--g---------------------tc-------------------- 240

G (G383) a----g-tact-atct--g--c-at--gtctg-c--g---------------------tc-------------------- 240

D (G91) ----cagtact-gtcc--g--c-at-----------a---------g-c--------ttct---------c--------- 240

D (G242) ----cagtact-gtcc--g--c-at-----------a---------g-c--------ttc----------c--------- 240

H (JIC15) ----cagtact-gtcc--g--c-a------------a-----------a--------ttc----------c--------- 240

H (CAR11) ----cagtact-gtcc--g--c-at-----------a-----------a--------ttc----------c--------- 240

I (G374) tg--cagtact-gtctcag----at---gtct--------------------------tc---------tc--------- 240

I (G375) tg--cagtact-gtctcag----at---gtct--------------------------tc---------tc--------- 240

I (JIC13) tg--cagtact-gtctcag----at---gtct--------------------------tc---------tc--------- 240

A (G180) GTCGCGTCGGTGAGATAGTTATCTTTACCTTTGTTTCAGTGCTAGGCCTTTACCTGCTTTGGCTTTGGGTGCTCAGAGAT 320

A (M34) ---------------------------------------------------------------------------a---- 320

Intron donor GT[7]

Intron[7]

T-Tracts[7]

Branch site[7]

Alternative branch site[7]

A (G191) ---------------------------------------------------------------------------a---- 320

A (KAR) ---------------------------------------------------------------------------a---- 320

A (G263) -------------------c--------t----------------------------------------------a---- 320

A (NAC1) ---------------------------------------------------------------------------a---- 320

C (G169) -------------------c-----------------g--------t----------------------------a---- 320

B (KEN) -------------------c-------------------------ct----------------------------a---- 320

E (JIC10) ------------------c---------t----------------ct------t----------------c--------- 320

E (G359) ------------------c------c-------------------ct------t----------------c--------- 320

E (G363) ------------------c--------------------------ct------t----------------c--------- 320

F (G364) -------------------c-------------------------ct----------------------------a---- 320

G (G384) -------------------c---------------------t---cg------t--------t-g----------a---- 320

G (G385) -------------------c------------------a--t---cg------t----------g----------a---- 320

G (G386) -------------------c---------------------t---cg------t--------t-g----------a---- 320

G (G383) ---------------c---c---------------------t---cg------t--------t-g----------a---- 320

D (G91) ------------------c----------------g----c----ct------t----------------c--------- 320

D (G242) ------------------c----------------g----c----ct------t-------------------------- 320

H (JIC15) ------------------c----------------g----t----ct-----------------------c--------- 320

H (CAR11) ------------------c----------------g-t--c----ct-----------------------c--------- 320

I (G374) ------------------c----------------g-t--t--t-ctt-g--------g-----------c--t------ 320

I (G375) ------------------c----------------g-t--t--t-ctt-g--------g-----------c--t------ 320

I (JIC13) ------------------c----------------g-t--t--g-ctt-g--------g-----------c--t------ 320

Intron acceptor AG[7]

A (G180) TGTATCTTACTTCTGAAGGCTCAGCGAGGTAGATCCACGGAGGAGCTGATATTTGGTCCTGGTGAGAGGCCAGCCGTCGC 400

A (M34) --------------------------------------------------------c----------------------- 400

A (G191) --------g----------------------------------------------------------------------- 400

A (KAR) --------------------------------------------------c----------------------------- 400

A (G263) --------------------------------------------------c----------------------------- 400

A (NAC1) --------------------------------------------------c----------------------------- 400

C (G169) --------------------------------------------------c----------------------------- 400

B (KEN) --------------------------------------------------c---------------------c------- 400

E (JIC10) ct------g-----------------------------------------c----------------------------- 400

E (G359) ct------g---------------------gt------------------c----------------------------- 400

E (G363) ct------g----------------------------------------------------------------------- 400

F (G364) -------------------------------------------------------------------------------- 400

G (G384) --c-----g--------------------------------------c-ct-----------------tt---------t 400

G (G385) --c-----g--------------------------------------c-cc-----------------tt---------t 400

G (G386) --c-----g--------------------------------------c-cc-----------------tt---------t 400

G (G383) --------g--------------------------------------c-cc-----------------tt---t-----t 400

D (G91) ct------g---g-t--------------ac-------c-----------c----------------------------- 400

D (G242) ct------g---g-t--------------ac-------------------c----------------------------- 400

H (JIC15) ct------g---g-t---------------c-------c-----------c--------------------cc------- 400

H (CAR11) ct------g---g-t---------------c-------c-----------c--------------------cc------- 400

I (G374) ct------------------------c---c-------------------c--------------------cc-t----- 400

I (G375) ct------------------------c---c-------------------c--------------------cc-t----- 400

I (JIC13) ct----------g-------------c---c-c--t--------------c--------------------cc------- 400

Movement protein stop codon

A (G180) TTGTGCCGACGGTTCTCGTCCTGTTCCAGATCCGTCTCCGTCCTGTCCACCGGGACCTAGACCTTTCGTGG-TCTAGAGC 480

A (M34) --c---------------c--c--------------------------g------------------------------- 480

A (G191) --c---------------ct----------------------g-----g------------------------------- 480

A (KAR) --c---------------ct-c--------------------------g------------------------------- 480

A (G263) --c---------------ct-c--------------------------g------------------------------- 480

A (NAC1) --c---------------ct-c--------------------------g------------------------------- 480

C (G169) --c---------------------------------g--c--------g------------------------------- 480

B (KEN) c-c---------------------------------g--c--------gt-----------------ac--g-------- 480

E (JIC10) cgc----------------------t-g--------g------------------------------------------- 480

E (G359) cgc----------------------t-g--------g----------------------------------g-------- 480

E (G363) cgc----------------------t-g--------g-----------------c------------------------- 480

F (G364) -gc---------------------------------g------------------------------ac----------- 480

G (G384) ccc----------------------g----------g-----------------------g--c--t------------- 480

G (G385) ccc----------------------g----------g-----------------------g--c--t------------- 480

G (G386) ccc----------------------g----------g-----------------------g--c--t------------- 480

G (G383) ccc------------------c---g----------g-----------------------g--c--t------------- 480

D (G91) ccc------------------------g--------------------g-----t-----g--a--t-----c------- 480

D (G242) ccc------------------------g--------------------g-----t-----g-----t-----c------- 480

H (JIC15) -cc-------------------c-c--g--------g--c--------g-----t-----g-----t-----c------- 480

H (CAR11) -cc----------c--------c-c--g--------g-----------g-----t-----g-----t-----c------- 480

I (G374) --c---------------------c--g-t------g----------------t------t--a---t---g-------- 480

I (G375) --c---------------------c--g-t------g----------------t------t--a--tt---g-------- 480

I (JIC13) --c------------------------g-t------g--t--t-----g----t------c-----t----g-------- 480

A (G180) ACTCAGCATGTCAGGAGCTTTGAAGCGTAAGCGTTCGGATGAGGTTGCCTGGAGTCGAAGGAAGCCCGTCAAGAAGCAAG 560

A (M34) ------------t--------------c---------------------------------------------------- 560

Coat protein start codon[5]

A (G191) ------------c--------------c---------------------------------------------------- 560

A (KAR) ------------c--------------c---------------------------------------------------- 560

A (G263) ------------c--------------c---------------------------------------------------- 560

A (NAC1) ------------c--------------c---------------------------------------------------- 560

C (G169) ------------t--------------c-------------------------------------------------ct- 560

B (KEN) ------------t--t-----------c--------t-------------------------g------g-------c-- 560

E (JIC10) -----t------c--------------g--------c--c-----------------------------g-------ct- 560

E (G359) -----t------t--t-----------c--------c--c-----------------------------g-------ct- 560

E (G363) -----t------c--------------c--------c--c-----------------------------g-------ct- 560

F (G364) ------------t------a------------------------------------------g------g-------ct- 560

G (G384) ------------g--g--g--------c-----------------------------------------g-------ct- 560

G (G385) ------------g--g-----------c-----------------------------------------g-------ct- 560

G (G386) ------------g--g-----------c-----------------------------------------g-------ct- 560

G (G383) ------------g--g-----------------------------------------------------g-------ct- 560

D (G91) t-----------c-----g--------c--------t--------g--------------------a-c--------c-- 560

D (G242) t-----------c--------------c--------t-----------------------------a-c--------c-- 560

H (JIC15) tt----------c--------------c--------------------------------------a-c--------c-- 560

H (CAR11) t-----------c--------------c--------------------------------------a-c--------c-- 560

I (G374) ----------g-c--g--g--------c---a-g--c-----------------------------t-c--------ct- 560

I (G375) ----------g-c--g--g--------c---a-g--c-----------------------------t-c--------ct- 560

I (JIC13) ----------g-c--g--g--------c---a-g--c-----------------------------t-c--------ct- 560

A (G180) -C-CCGG-GTTCCCCCTGCCC------GGGCTGGCCCCTCTGTCAGGAGAGGACTCCCTGCCCTTCAGATCCAGACGCTC 640

A (M34) ----------c--g---c----------------------c--------------------------------------- 640

A (G191) -----------------c-------------------a--c--------------------------------------- 640

A (KAR) a-a--------------c---c---------------------------------------------------------t 640

A (G263) -----------------c-------------------------------------------------------------t 640

A (NAC1) -----------------c-------------------------------------------------------------t 640

C (G169) ------cc-g------ac---c---gc-------------------------t---------t-g-----t-----ct-g 640

B (KEN) -t----cc---g----ac-t-c---gc--------------------------------t----------------ct-g 640

E (JIC10) ------tc-g------ac-g-c---gc------ct--------------g--t------t----g-----t-----ct-g 640

E (G359) ------cc-g------ac-g-c---gc------ct--------------g--t------t----g-----t-----tt-a 640

E (G363) ------tc-g------gc-g-c---gc------ct--------------g--t------t----g-----t-----ct-g 640

F (G364) -------c-c---t--cc-g-c---cc-------------------------------------------t-----ct-g 640

G (G384) -------a-g------ac-a-ctcccc--------t-g--ctc--a---gac------------------a-----ct-a 640

G (G385) -------a-g------ac-t-ctgccc--------t-g--ctc--a---gac------------------a-----ct-a 640

G (G386) -------a-g------ac-a-ctcccc--------t-g--ctc--a---gac------------------a-----ct-a 640

G (G383) -------a-g------ac-a-ctcccc--------t-g--ctc--a---gac------------------a-----ct-a 640

D (G91) ------tcag------ac-------------------g--c--------g--------------c-----t-----ct-g 640

D (G242) ------tcag------ac-------------------g--c--------g--------------c-----t-----ct-g 640

H (JIC15) ------tcag------ac-------------------g--c-----------------------------a-----ct-g 640

H (CAR11) ------tcag------cc---c---gc----------g--c--------g--------------------a-----ct-g 640

I (G374) -------c-c---t---c-g-----------------a--c--------g--------------------t---t-ct-g 640

I (G375) -------c-cc--t---c-g-----------------a--c--------g--------------c-----t---t-ct-g 640

I (JIC13) -------c-cc------c-g-----------------a--c--------g--------------------------ct-g 640

A (G180) ATAGCGGCTGGTGACACTATGATCACGGTGCCGTCCGGCGGCATCTGCAGTCTGATTGGCACGTATGCCCGGGGCTCTGA 720

A (M34) -----------------------------------t--------------------------------------t----- 720

A (G191) -------------------------------------------------------------------------------- 720

A (KAR) -cg----------------------------------------------------------------------------- 720

A (G263) -cg----------------------------------------------------------------------------- 720

A (NAC1) -cg----------------------------------------------------------------------------- 720

C (G169) g-g--t--c--------c--------t--------a-----------------------------------------a-g 720

B (KEN) g-g--t--c-----------------t-----------a----------------------------------------g 720

E (JIC10) g-g--t--c-----------------t-----------a--------------------------------------c-g 720

E (G359) g-g--t--c-----------------t-----------a--------------------------------------c-g 720

E (G363) g-g--t--c-----------------a--------t-----------------------------------------c-g 720

F (G364) g-g--t--c-----------------t--------a--a-----------------------------------t----g 720

G (G384) g-g--t--c-----------------t-----------a-----------------------------------g--c-g 720

G (G385) g-g--t--c-----------------t--------t--a----------------------------g------g--c-g 720

G (G386) g-g--t--c-----------------t-----------a-----------------------------------g--c-g 720

G (G383) g-g--t--c-----------------t-----------a-----------------------------------g--c-g 720

D (G91) g-g--t--c-----t-----------t--------a------------------c-------c--------c--a----- 720

D (G242) g-g--t--c-----t-----------t--------a------------------c-------c--------c--a----- 720

H (JIC15) g-g--t--c-----g-----------t--------a--t--------t------c-------c--------c--g----- 720

H (CAR11) g-g--t--c-----g-----------a---------------------------c-------c--ct----t--g----- 720

I (G374) g-g--t--c-----g-----------------taa-------------tc--c-----tg--c--ct----t--g--c-- 720

I (G375) g-g--t--c-----g-----------------taa-------------tc--c-----tg--c--ct----t--g--c-- 720

I (JIC13) g-g--t--c-----------------a---g-caa------t------tc--c-c---tg--c--ct-t--t--g--c-- 720

A (G180) TGAAGGTAACCGCCACACCAACGAGACTCTGACGTACAAGGTTGCGCTGGACTACCACTTCGTTGCTACTGCTGCGGCCT 800

A (M34) -------------------------------------------------------------------------------- 800

A (G191) ---------------------------------------------------------------------c---------- 800

A (KAR) -------------------------------------------------------------------------------- 800

A (G263) ---------------------------------------------------t---------------------------- 800

A (NAC1) -------------------------------------------------------------------------------- 800

C (G169) -------g-a-----------------------------------t-----------------------c--c------- 800

B (KEN) -------g-a--------------------------------c--t--------------------c--c-----t--g- 800

E (JIC10) ---g---g-a--------------------------------c--t--------------------c--c-----t--g- 800

E (G359) ---g---g-a--------------------------------c--t--------------------c--c-----t--g- 800

E (G363) ---g---g-a-----t--------------------------c--t--------------------c--c-----t--g- 800

F (G364) ------ag-a--t--------------------------------t--------------------ct--t----t--g- 800

G (G384) ---g---g-a-----------------------------------c-----------t--t--a-----c--c--t--g- 800

G (G385) ---g---g-a-----------------------------------c-----------t--t--a-----c--c--t--g- 800

G (G386) ---g---g-a-----------------------------------c-----------t--t--a-----c--c--t--g- 800

G (G383) ---g---g-a-----------------------------------c-----------t--t--a-----c--c--t--g- 800

D (G91) ------a-----------------------a---------------t-a-----------t-----c--c-----t--g- 800

D (G242) ------a-----t-----------------a---------------t-a-----------t-----c--c-----t--g- 800

H (JIC15) ------a-----------------------a---------------t-a-----------------c--c-----t--t- 800

H (CAR11) ------a-----------------------a---------------t-a-----------------c--c-----t---- 800

I (G374) ---g-----------t------------------------a-a--ct----------t--t--gctgt-ct-g--t---- 800

I (G375) ---g-----------t------------------------a-a--ct----------t--t--gctgt-ct-g--t---- 800

I (JIC13) ---g-----------t------------------------a-a--ct----------t--t--gttgt-ct-g------- 800

A (G180) GCAAGTACTCCAGCATTGGAGTGGGTGTCATGTGGTTGGTGTACGATGCTCAGCCGACCGGCAATTCACCGGAGGTGAAG 880

A (M34) -------------------------------------------t-----g------------------------------ 880

A (G191) -------------------------------------------t-----g------------------------------ 880

A (KAR) -------------------------------------------t-----g------------------------------ 880

A (G263) -------------------------------------------t-----g------------------------------ 880

A (NAC1) --cgt--------------------------------------t-----g------------------------------ 880

C (G169) ----------------------c-----gtgc-----------------g-----t-------c-g-c---act---c-- 880

B (KEN) -t--a---ag---t-----ca-t--a--gtgc-----------------g-----t-------c-g-c---acc--cc-a 880

E (JIC10) -t--a---ag---t-----ta-t--a--ttgc-----------------g-----t-------c-g-c---acc--cc-a 880

E (G359) -t--a---ag---t-----ca-t--a--ttgc-----------------g-----t-------c-g-c---acc--cc-a 880

E (G363) -t--a---ag---t-----ga-t--a--ttgc-----------------g-----t-------c-g-c---acc--cc-a 880

F (G364) -t--a---ag---t-----t--t--a--ttgc-----------------g-----t-------c-g-c---acc--cc-a 880

G (G384) -t--a---ag------c--ga-c--a--gtgc-----------------c-----t-------c-g-c---acc--cc-a 880

G (G385) -t--a---ag------c--ga-c--a--gtgc-----------------c-----t-------c-g-c---acc--cc-a 880

G (G386) -t--a---ag------c--ga-c--a--gtgc-----------------c-----t-------c-g-c---acc--cc-a 880

G (G383) -t--a---ag------c--ga-c--a--gtgc-----------------c-----t-------c-g-c---acc--cc-a 880

D (G91) -t--a---ag---t-----t--t--a--gtgc-----------------g-----tt--------g-c------------ 880

D (G242) -t--a---ag---t-----t--t--a--gtgc-----------------g-----tt--------g-c------------ 880

H (JIC15) -t--a---ag---t-----t--t--a--gtgc-----------------g-----tt--------g-c----cc------ 880

H (CAR11) -t--a---ag---t-----t--t--a--gtgc-----------------g-----tt--------g-c----cc------ 880

I (G374) -t-----tg----tcgc-ct--t--c--gg-c--------c--t-----g---------------g-c---c-a------ 880

I (G375) -t-----tg----tcgc-ct--t--c--gg-c--------c--t-----g---------------g-c---c-a------ 880

I (JIC13) -----c-tg----tcgc-ct--t--c--gg-c--------c--t-----g---------------g-c---c-a------ 880

A (G180) GACATCTTCCCTCACTCCGATACGCTCTCAGCGTTCCCCTACACTTGGAAGGTTGGCAGGGAGGTCTGCCATCGCTTCGT 960

A (M34) --------------------c----------------------------------------------------------- 960

A (G191) -------------------------------------------------------------------------------- 960

A (KAR) --------------------------------------g----------------------------------------- 960

A (G263) --------------------c----------------------------------------------------------- 960

A (NAC1) --------------------c--------------------------------------------g-------------- 960

C (G169) --------t--g---c-gac------tg-c--a-----g-----c--------g--g----------------------- 960

B (KEN) -----t--t------c-g-c---t--------c--t--------c--------g--g----------------------- 960

E (JIC10) -----t--t--a---c-g-c---t--------a--t--------c--------g--g----------------------- 960

E (G359) -----t--t--a---c-g-c---t--------a--t--------c--------g--g----------------------- 960

E (G363) --t--t--t--a---c-g-cc--t--------------------c--------g--g----------------------- 960

F (G364) -----a--t--g---c-g-c---c--------c--t--------c--------g--g----------------------- 960

G (G384) --t--a--t--g---c-g--c--t--g--c--t-----g-----c--------g--g----------------------- 960

G (G385) --t--a--t--g---c-g--c--t--g--c--t-----g-----c--------g--g----------------------- 960

G (G386) --t--a--t--g---c-g--c--t--g--c--t-----g--t--c--------g--g----------------------- 960

G (G383) --t--a--t--g---c-g--c--t--g--c--t-----g--t--c--------g--g----------------------- 960

D (G91) --t-----------tgga--------t--c--c-----------------------g----------------------- 960

D (G242) --t-----------tgga--------t--c--c-----------------------g----------------------- 960

H (JIC15) -----------g--tgga--c--------c--c-----------------------g----------------------- 960

H (CAR11) -----------g--tgga--c--------c--c-----------------a-----gc---------------------- 960

I (G374) ---------------gg----g----g--c--c-----g-----------------t----------------------- 960

I (G375) ---------------gg----g----g--c--c-----g-----------------t----------------------- 960

I (JIC13) ---------------gg---------g--c--c-----g-----------------g--------g-------------- 960

A (G180) TGTTAAACGGCGCTGGTGCTTCACCATGGAGACTAACGGCCGGATCGGTTCAGATGTTCCTCCGGCCAATACCGCTTGGC 1040

A (M34) -------------------------------------------------------a-c---------------------- 1040

A (G191) ---------------------------------------------------g---a-c---------------------- 1040

A (KAR) ---------------------------------------------------------g---------------------- 1040

A (G263) ------------------------------------------------------c------------------------- 1040

A (NAC1) ---------------------t--------------------------------c------------------------- 1040

C (G169) ------------------------------------------------c--------g--a---t----c--a--g---- 1040

B (KEN) ---------------------t---------------------------------ac---c---t----cgtg------- 1040

E (JIC10) ---------------------t--------------------c--------g---aca--c---t----cgtg------- 1040

E (G359) ---------------------t-----------------------------g---acc--c---t----cgtg------- 1040

E (G363) ---------------------t--------------------c--------g---aca--c---t----cgtg------- 1040

F (G364) -------------------------------------------------------ac---c---t----cgtg------- 1040

G (G384) c-----ga--a-g---------------tcc--c--t---a-------g--t--c--c-----tt----c--a------- 1040

G (G385) c-----ga--a-g---------------tcc--c--t---a-------g--t--c--c-----tt----c--a------- 1040

G (G386) c-----ga--a-g---------------tcc--c--t---a-------g--t--c--c------t----c--a------- 1040

G (G383) c-----ga--a-g---------------tcc--c--t---a-------g--t--c--c------t----c--a------- 1040

D (G91) g--g--g--a------acg--t-----------cg----t--c--t--g--g-----c--------t---g--t------ 1040

D (G242) g--g--g--a------acg--------------cg----t--c--t--g--g-----c--------t---g--t------ 1040

H (JIC15) g--c--ga-a------acg--------------cg----t--c--t--g--g-----c------c-tg--g-tt------ 1040

H (CAR11) g--c--ga-a------acg--------------cg----t--t--t--g--g-----c------c-tg--g-tt------ 1040

I (G374) g-----g-----g---act-----g-------gc------a-a--------g--caca-----t-ag---g-t------a 1040

I (G375) g-----g-----g---act-----g-------gc------a-a--------g--caca-----t-ag---g-t------a 1040

I (JIC13) g--g--g-----g---act-----g-------gc------a-a--------g---acg-----t--g---g-t------a 1040

A (G180) CGCCTTGCAAGAAGGACATCTACTTCCACAAGTTCTGCACGGGACTCGGCGTGAAGACGGAGTGGAAGAACGTTACAGAC 1120

A (M34) ----a--t---------c-------------------------------------------------------g------ 1120

A (G191) ----a--t---------c-------------------------------------------------------g------ 1120

A (KAR) -------------------------------------------------------------------------------- 1120

A (G263) -------------------------------------------------------------------------------- 1120

A (NAC1) -----------------c-------------------------------------------------------g------ 1120

C (G169) -c--a--t---------------------------a-------ct-g--------------------------------- 1120

B (KEN) -a--c--------------------------------------ct-g--------------------------------- 1120

E (JIC10) -a--c--------------------------------------ct-g--------------------------------- 1120

E (G359) -a--c--------------------------------------ct-g--------------------------------- 1120

E (G363) -a--c--------------------------------------ct-g--------------------------------- 1120

F (G364) -a--c--------------t-----------------------ct-g--------------------------c------ 1120

G (G384) -c--c--t---------t-g------------------------t-g--a-----------------------c------ 1120

G (G385) -c--c--t---------c-g------------------------t-g--a-----------------------c------ 1120

G (G386) -c--c--t---------t-g------------------------t-g--a--------c--------------c------ 1120

G (G383) -c--a--t---------c-g------------------------t-a--a-----------------------c------ 1120

D (G91) ----a------cgctc------------------------t--------a------------------------------ 1120

D (G242) ----g------cgctc---a--------------------t--------a------------------------------ 1120

H (JIC15) ----a------cgctct-----------------------c--g-----g-----------------------c------ 1120

H (CAR11) ----c------cgctc------------------------c--g-----g-----------------------c------ 1120

I (G374) ----cgtg---cgctc------------------------t--------g-----------------------g------ 1120

I (G375) ----cgtg---cgttc------------------------t--------g-----------------------g------ 1120

I (JIC13) ----cgtg---cgctc------------------------t--------g-----------------------g------ 1120

A (G180) GGGAAGGTCGGCGCTATTAAGAAGGGCGCCTTGTACATAGTCATTGCGCCTGGAAACGGGCTTGAGTTTACGGTTCACGG 1200

A (M34) -------------------------------------------------------------------------------- 1200

A (G191) --------------------------------------t-----------------------c----------------- 1200

A (KAR) --------------------------------------t----------------------------------------- 1200

A (G263) --------------------------------------t----------------------------------------- 1200

A (NAC1) -------------------------------------------------------------------------------- 1200

C (G169) -------a------c--c----------g---------------------c--g--------c-----c---tg------ 1200

B (KEN) -------a------c--c----------g---c-----------------c--g--------a-----c---tg------ 1200

E (JIC10) -------a------c--c----------g---c-----------------c--g-----c--a-----c---tg------ 1200

E (G359) -------a------c--c----------g---c-----------------c--c-----c--c-----c---tg------ 1200

E (G363) -------a------c--c----------g---c-----------------c--c-----c--c-----c---tg------ 1200

F (G364) -------a------c--c----------g------------------------g--------c----------------- 1200

G (G384) -------a------c-------------g------------------a--g--c--t--c--c-----c----------- 1200

G (G385) -------a------c-------------g------------------a--g--c--t--c--c-----c----------- 1200

G (G386) -------a------c-------------g---------t--------a--g--c--t--c--c-----c----------- 1200

G (G383) -------a------c--c----------g------------------a--g--c--t--c--c----------------- 1200

D (G91) --------------c-----------------------t--tc----c--g--c-----c-------------------- 1200

D (G242) --------------c-----------------------t--tc----c--g--c-----c-------------------- 1200

H (JIC15) --------------c--c-----------------t--ta-tc----c--g--c-----c---------------a---- 1200

H (CAR11) --------------c-----------------------ta-tc----c--g--c-----c-------------------- 1200

I (G374) --tggt--------c--------------------t--t--a-----t--g--c--t--c--c--c--c----------- 1200

I (G375) --tggt--------c--------------------t--t--a-----t--g--c-----c--g--c--c----------- 1200

I (JIC13) --tggt--------c-----------------------t--a-----t--g--c-----c--g--c--c----c------ 1200

A (G180) CCAGTGCCGTCTGTACTTTAAGTCAGTTGGTAATCAGTGATTACCACATCA-ATTAATAAAAC-AAGTT-TTAT-TCA-- 1280

Coat protein stop codon[5]

Virion sense gene polyadenylation signal[5]

Complementary sense gene polyadenylation signal[5]

A (M34) -------------------------------------------------------------------------------- 1280

A (G191) ----------------------------------------------t--------------------------------- 1280

A (KAR) -------------------------------------------------------------------------------- 1280

A (G263) -------------------------------------------------------------------------------- 1280

A (NAC1) ------------------c------------------------------------------------------------- 1280

C (G169) ------------------------t-----g-----------c----t---at-c-----------------------ta 1280

B (KEN) ------------------------t-----g-----------c-ac-gca--t-------------------------t- 1280

E (JIC10) ------------------------t-----g-----------c----t-a-tt-------------------------tt 1280

E (G359) ------------------------------g-----------c------a-tt-------------------------tt 1280

E (G363) ------------------------------g-----------c----t-a-tt-------------------------tt 1280

F (G364) ------------------------------g-----------c----t-t-at-------------------------ta 1280

G (G384) ------------------c-----------g-----------c-----ca-ata--------a-t-a-----------tt 1280

G (G385) ---------------t--c--------c--g-----------c-----ca-ata--------a-t-a-----------tt 1280

G (G386) ------------------c-----------g-----------c-----a--at---------a-t-a-----------tt 1280

G (G383) ------------------c-----------g-----------c------a-ata--------a-t-a-----------tt 1280

D (G91) ---agc----t-------------t-----g-----------c----t-a--t-----------tt------------ta 1280

D (G242) ---agc----t-------c-----t-----g-----------c----t-a--t-----------tt------------ta 1280

H (JIC15) ---agc----t----t--------t-----g-----------c----t----------------tt------------aa 1280

H (CAR11) ---agc---at-------c-----t-----g-----------c----t----------------tt--------t---a- 1280

I (G374) ---------------------------c--g-----------c---t-aatttaat-a---ct-tgt-------tg--ag 1280

I (G375) ------------------------------g-----------c-----aa-ttaat-a---ct-tgt-------tc--at 1280

I (JIC13) ---------------------------c--g-----------c------t--ta--------actt---t----tc--ga 1280

Complimentary strand origin of replication[8]

A (G180) AAG-AGCG--------AAGCTCA-TACATTACATAGTCAGCAGATATGCTGACAGAAAA-CACACACATAGTGCAGCCTC 1360

A (M34) -g-------------------------------------------g---------------------------------- 1360

A (G191) -------------------------------------------------------------------------------- 1360

A (KAR) -------------------------------------------------------------------------------- 1360

A (G263) -------------------------------------------------------------------------------- 1360

A (NAC1) -------------------------------------------------------------------------------- 1360

C (G169) ct---a----------g--t------------------------------------------------------------ 1360

B (KEN) -t---a----------g--t------------------g----t--a-ac---ga----a-------------------- 1360

E (JIC10) c----a-------------t------------------t------t---a---ga------------------------- 1360

E (G359) c----a-------------t--g---------------t------t---a---ga------------------------- 1360

E (G363) c----a-------------t--g---------------t------t---a---ga------------------------- 1360

F (G364) c----a----------g--t------------------g----t--a--c---ga------------------------- 1360

G (G384) c----a----------g--g--t---------------g------ta--c---ga----a-------------------- 1360

G (G385) c----a----------g--t--t---------------g------ta--c---ga----a-------------------- 1360

G (G386) -----a----------g--t--t---------------g------ta--c---ga----a-------------------- 1360

G (G383) c----a----------g--t--t---------------g------ta--c---ga----a-------------------- 1360

D (G91) ct---a-------------t------------------g------t---c---ga------------------------- 1360

D (G242) ct---a-------------t------------------g------t---c---ga------------------------- 1360

H (JIC15) ct---a-------------t--------a---------g------t---c---gac------------------------ 1360

H (CAR11) ct---a-------------t------------------g------t---c---gac------------------------ 1360

I (G374) ccc----atttaaaatgc-ta--t--------------g------t---c---ga------------------------- 1360

I (G375) ccc----atttaaaatgc-ta--t--------------g------t---c---ga------------------------- 1360

I (JIC13) c-tc---attgaaaatgc-ta--t--------------g----t-t---c---ga------------------------- 1360

Sequence conserved in MSV subgenomics

Replication associated protein stop codon

A (G180) GGGCTAAAGACCGAGTCTCAAACGACCCAACTTAAAAACAAATCAAAACAAGCATGATAT-TATTAAATATGCAGCCGCC 1440

A (M34) -------------------------------------------------------------------------------- 1440

A (G191) -------------------------------------------------------------------------------- 1440

A (KAR) -------------------------------------------------------------------------------- 1440

A (G263) -------------------------------------------------------------------------------- 1440

A (NAC1) -------------------------------------------------------------------------------- 1440

C (G169) --------------------------------------------------------------------cc---------- 1440

B (KEN) -----------------------------------------------------------------t--a----t------ 1440

E (JIC10) ---------------------------------------------------a----------------cc---------- 1440

E (G359) ---------------------------------------------------a----------------cc---------- 1440

E (G363) ---------------------------------------------------a----------------cc---------- 1440

F (G364) --------------------------------------------------------------------ac---------t 1440

G (G384) ----------------------------c-aa-t-----------g----------------------ac---------- 1440

G (G385) ----------------------------c-aa-t-----------g----------------------ac---------- 1440

G (G386) ----------------------------c-aa-t-----------g----------------------ac---------- 1440

G (G383) ----------------------------c-aa-t-----------g----------------------ac---------- 1440

D (G91) --------------------------------------------------------------------at---------- 1440

D (G242) --------------------------------------------------------------------at---------- 1440

H (JIC15) ------------------------------------c-----------a---------t---------ac---------t 1440

H (CAR11) ------------------------------------c-----------a---------t---------ac---------t 1440

I (G374) ----------------------------c-aa-t----a------g--g-----a-----a-------at---------- 1440

I (G375) ----------------------------c-aa-t----a------g--g-----a-----a-------at---------- 1440

I (JIC13) ----------------------------c----------------g--g-----a-----a-------at---------- 1440

A (G180) GGCTTAAGCA-----GGAGTGAACCACTTCTCTCCTGCCTGCATTACGTAGATTTCGCAGTTCGCGTAGAAGTAGTCATA 1520

Replication associated protein stop codon

A (M34) --------------------------t-----------------------------------t--------------g-- 1520

A (G191) -----------------------------------------------------c--------t----------------- 1520

A (KAR) --------------------------------------------------------------t----------------- 1520

A (G263) --------------------------------------------------------------t----------------- 1520

A (NAC1) --------------------------t-----------------------------------t----------------- 1520

C (G169) ------t----------c---t----------c--c--------c-----t-----------t----------------- 1520

B (KEN) -----ctc--ctgtctt---ag----t--------a-g-a-g-gg-t---ttgg--------t--c-c------c--cac 1520

E (JIC10) ---------------------t----------------t--------------c--------t-----------c--g-- 1520

E (G359) ---------------------t----------------t--------------c--------t-----------c--g-- 1520

E (G363) ---------------------t----------------t--------------c--------t-----------c--g-- 1520

F (G364) -c---c-t-tctgtct---aag-------------a-a-a---g-gt---ctgg--------a--c-ca------ctcag 1520

G (G384) -----c-t-tctgtct---aag----t--------a-a-a---g-gt---ctgg-----------c-c-------ctc-g 1520

G (G385) -----c-t-tctgtcc---aag----t--------g-a-a---g-gt---ctgg-----------c-c-------ctc-g 1520

G (G386) -----c-t-tctgtcc---aag----t--------g-a-a---g-gt---ctgg-----------c-c-------ctc-g 1520

G (G383) -----c-t-tctgtct---aag----t--------a-a-a---g-gt---ctgg-----------c-c-------ctc-g 1520

D (G91) -----c-c-tctgtct---aag-------------a-a-a---gg-t---ctgg--------t--c-c-------ctcag 1520

D (G242) -----c-c-tctgtct---aag-------------a-a-a---gg-t---ctgg--------t--c-c-------ctcag 1520

H (JIC15) ------t--c-----------t-------------g--------------------------g----------------- 1520

H (CAR11) ------t--c-----------t-------------g--t-----------------------g----------------- 1520

I (G374) ------g-ag-------g---t-------t--c--ct----------------c--------tt----------c----- 1520

I (G375) ------g-ag-------g---t-------t--c--ct----------------c--------tt----------c----- 1520

I (JIC13) ------g-ag-------g---t----------c--ct----------------c--------tt----------c----- 1520

RepA stop codon

A (G180) CTGCGCCGGAGTCATGTCCTTCAGCCAGTCCTCATCCTCGTTGGCGAGGATGATAGTTGGTATGCTCTTTGAGGCTACCT 1600

A (M34) ------------------------------------------------------------a--------------c---- 1600

A (G191) ---------------------------------------------------------------------------c---- 1600

A (KAR) ---------------------------------------------------------------------------c---- 1600

A (G263) ---------------------------------------------------------------------------c---- 1600

A (NAC1) ---------------------------------------------------------------------------c---- 1600

C (G169) ---t-a---t----gc--t-----------------------------------g--a--a--------ctt-------- 1600

B (KEN) g-----------------------------------------------------------a--------g--t------- 1600

E (JIC10) ---g-----t----gt-----------------------------------t--t--a--g--------ctt---c---- 1600

E (G359) ---g-----t----gt-----------------------------------t--t--a-------c---ctt---g-a-c 1600

E (G363) ---g-----t----gt-----------------------------------t--t--a--g--------ctt---g---- 1600

F (G364) ------t-----------------------------t-----------------t--a--a-----t--ctt-------- 1600

G (G384) -------------------c----------------t-----------------t--g--g--------ccg---c---- 1600

G (G385) -------------------c----------------t-----------------t--g-----------ccg---c---- 1600

G (G386) -------------------c----------------t-----------------t--g--g--------ccg---c---- 1600

G (G383) -------------------c----------------t-----------------t--g--g--a-----ccg---c---- 1600

D (G91) -------------------c----------------------------------g-----a--------cttt------- 1600

D (G242) -------------------c----------------------------------t-----a--------cttt------- 1600

H (JIC15) ---------c-----t--------------------------------------t-----a--------cttt------- 1600

H (CAR11) ---------c-------------t------------------------------t-----a--------cttt--c---- 1600

I (G374) -----------------------t---a----------t---------------t--g--g--------ctt------t- 1600

I (G375) -----------------------t---a----------t------------t--t--g--g--------ctt------t- 1600

I (JIC13) -----------------------t--------------t---------------c--g--g--------ctt-------- 1600

RepA stop codon

A (G180) TCCGTCTCTTCCCGTACTTCGGGTTCACTATGTAGTCTTTTTGACAGCCGACGAGCTGCTTCCAGCACGGACAGAACTTG 1680

A (M34) -------------------------------------c------------------------------------------ 1680

A (G191) -------------------------------------------g------------------------------------ 1680

A (KAR) -------------------------------------------------------------------------------- 1680

A (G263) -------------------------------------------------------------------------------- 1680

A (NAC1) -------------------------------------c------------------------------------------ 1680

C (G169) ----c-g------------t--a--------------gg-c-----a--------------------------------- 1680

B (KEN) ----g-gt-----------g--a--g-----------------g-----------tc-t----t---------------- 1680

E (JIC10) ----------g-----------a--t------------g----g------------------------------------ 1680

E (G359) -g-----t-----------g--a--g-----------cg----------------------------------------- 1680

E (G363) -g-----------------g--a--g-----------cg----------------------------------------- 1680

F (G364) ----------g-----t--g--a--g-----------ag----------t--------------------g--------- 1680

G (G384) ------g------------g--a--g-----------gg----------t--------------------g--------- 1680

G (G385) ----c-g---------t--g--a--g-----------gg----------t--------------------g--------- 1680

G (G386) ------g------------g--a--g-----------gg----------t--------------------g--------- 1680

G (G383) ------g---------------a--g-----------gg----------t--------------------g--------- 1680

D (G91) ----g-gt-----------g--a--g------------g----g------------------------------------ 1680

D (G242) ----g-gt-----------g--a--g------------g----g------------------------------------ 1680

H (JIC15) ----g-gt-----------g--a--g------------g----g------------------------------------ 1680

H (CAR11) ----g-g------------g--a--g------------g----g------------------------------------ 1680

I (G374) -g--g-gt-----------------g--g--------------------------------------------------- 1680

I (G375) -g----gt-----------------g--g--------------------------------------------------- 1680

I (JIC13) ------gt-----------g-----g--g--------------------------------------------------- 1680

A (G180) AAGGGAATGTCGTCAACGACATTGTAGACAGCTTCTTCATCATATGAAGACCAGTCGATATTATTCTGCCAGTAGTTGTG 1760

A (M34) --------------------------------------------------------------g----------------- 1760

A (G191) -----------------------------------------------------------------t-------------- 1760

A (KAR) --------------c-----------cg-------------g-----------------g-------------------- 1760

A (G263) --------------c-----------cg-------------g-------------------------------------- 1760

A (NAC1) -----------------------------------------g-------------------------------------- 1760

C (G169) ----------------------------g--------------------------------------------------- 1760

B (KEN) --------------------------ctg---------------------------------g--t-------------- 1760

E (JIC10) ----------------t-----------g-----------------------------c---g----------------- 1760

E (G359) ----------------t-----------g-----------------------------c---g----------------- 1760

E (G363) ----------------t-----------g-----------------------------c---g----------------- 1760

F (G364) --------------------------ctg-----------------------------c--------------------- 1760

G (G384) ----------------------------g------------g--------------------g----------------- 1760

G (G385) ----------------------------g---------------------------------g--------a-------- 1760

G (G386) ----------------------------g---------------------------------g----------------- 1760

G (G383) ----------------------------g---------------------------------g----------------- 1760

D (G91) ----------------------------g---------------------------a-----g--t-------------- 1760

D (G242) ----------------------------g---------------------------a-----g--t-------------- 1760

H (JIC15) ----------------------------g-----------------------------c---g--t-------------- 1760

H (CAR11) ----------------------------g---------------------------------g----------------- 1760

I (G374) --------------g-------------g------------g--------------------g--------a-----a-- 1760

I (G375) --------------g-------------g---------------------------------g--------a-----a-- 1760

I (JIC13) --------------g-------------g---------------------------------g--------a-------- 1760

A (G180) TCGTCCGAGGCTTCTGGCCCAGGATGTTTTACCTGTCCGTGTTGGGCCGACGATGTAGAGGCTGCGCTTTCTTGCTCCTG 1840

A (M34) c------------------------------------------------------------------------------- 1840

A (G191) -------------------------------------------------------------------------------- 1840

A (KAR) c--------------------------------------------------------------t---------------- 1840

A (G263) ------a--------------------------------------------------------t---------------- 1840

A (NAC1) -------------------------------------------------------------------------------- 1840

C (G169) c--------------------------------------------------------------tgt-------------- 1840

B (KEN) ---------------a---------------------------------ca------------t-t-------t------ 1840

E (JIC10) ---------------a-----------------------------------------------tgt-------------- 1840

E (G359) ---------------a-----------------------------------------------tgt------------c- 1840

E (G363) ---------------a-----------------------------------------------t-t-------------- 1840

F (G364) ---------------------a-t---c--------------------------------------t-------t----- 1840

G (G384) --tc----------g--------ta-----g--g--------------------------------t-------t----- 1840

G (G385) --tc----------g--------ta-----g--g--------------------------------t-------t----- 1840

G (G386) --tc----------g--------ta-----g--g--------------------------------t-------t----- 1840

G (G383) --tc----------g--------ta-----g--g--------------------------------t-------t----- 1840

D (G91) --t--------------------t------------------------------------------t-------t----- 1840

D (G242) --t--------------------tg-----------------------------------------t-------t----- 1840

H (JIC15) g-----------------------------t--------------------------------tgt-------------- 1840

H (CAR11) g-----------------------------t--------------------------------t-t-------------- 1840

I (G374) ---------------------t-----------------------------------------t-t-------------- 1840

I (G375) ---------------------t-----------------------------------------t-t-------------- 1840

I (JIC13) ---------------------t-----------------------------------------t-t--------a----- 1840

Branch site[9]

Intron[9]

Intron acceptor AG[9]

T-Tracts[9]

A (G180) GAGTAATCTGGTTGTTTCAGACAACCATTCTAAGTCAGCTTTGGCTTGCTCTAGGCTTAAGCAGCTAGGTTCAAGGAGCA 1920

A (M34) ---------------------------------------------c---------------------------------- 1920

A (G191) -------------------------------------------------------------------------------- 1920

A (KAR) --a----------------------------------------------------------------------------- 1920

A (G263) --a----------------------------------------------------------------------------- 1920

A (NAC1) -------------------------------------------------------------------------------- 1920

C (G169) ---------a----------ca-g---a--------------t------------------------------------- 1920

B (KEN) ---c-t---------a---tc------------------aa-t--c--t-----------------------t-----tt 1920

E (JIC10) ---------a----------c--g---g--------------t--------------------------------a---- 1920

E (G359) ---------a----------c--g---g--------------t-----------------------------g--a---- 1920

E (G363) ---------a----------c--g---g--------------t--------------------------------a---- 1920

F (G364) ---c-t---------------------a--------------t-----t-----------------t-----t------- 1920

G (G384) ---c-t---------------------------------c-----c--t-----------------------t-a----- 1920

G (G385) --ac-t---------------------------------c-----c--t-----------------------t-a----- 1920

G (G386) ---c-t---------------------------------c-----c--t-----------------------t-a----- 1920

G (G383) ---c-t---------------------------------c-----c--------------------------t-a----- 1920

D (G91) ---c-t--------------c---------------------t-----t-----------------------t-a----- 1920

D (G242) ---c-t------------------------------------t-----t-----a-----------------t-a----- 1920

H (JIC15) --------------------c------g--------------t-----------------------------t------- 1920

H (CAR11) -----c--------------c------g-g------------t-----------------------------t------- 1920

I (G374) --a------a--a--------g-g-----gc--------aa-t--c--t---c-------------------t------- 1920

I (G375) --a------a--a--------g-g-----gc--------aa-t--c--t---c-------------------t------- 1920

I (JIC13) --ag-gc--a--a-----g--g-g-----gc--------aa-t--c--t---c-------------------t-a----- 1920

A (G180) TGTATGCTTTAGGACTGACCTGGTAGATGTTAGGCTCAAGCCAGTCCTGGAGTGTTTCGTTGCAGAGGAGGTCAGGATCA 2000

Intron donor GT[9]

A (M34) ----------------------------------------------------------------------------g--- 2000

A (G191) -------------------------------------------------------------------------------- 2000

A (KAR) ----------------------------------------------------------------------------g--- 2000

A (G263) ----------------------------------------------------------------------------g--- 2000

A (NAC1) -------------------------------------------------------------------------------- 2000

C (G169) -------------------------------------------------------------------------t--g--t 2000

B (KEN) ----a----g------------a--t-----t----tt----------ca-t---c--a------t--------tat--- 2000

E (JIC10) -------------------------------t--------------------a--------------------t--t--- 2000

E (G359) -------------------------------c--------------------a--------------------t--g--- 2000

E (G363) -------------------------------t--------------------a--------------------t--g--- 2000

F (G364) ----a-----g--------------t-----t-----c---------c---t---c--a------t-------ta-c--- 2000

G (G384) ----------g---a----------t-----c-----c---------c---ta--c--a------t-------ga-t--g 2000

G (G385) ----------g--------------t-----c-----c---------c---ta--c--a------t-------ga-t--g 2000

G (G386) ----------g--------------t-----c-----c---------c---ta--c--a------t-------ga-t--g 2000

G (G383) ----------g--------------t-----c-----c---------c---ta--c--a------t-------ga-t--g 2000

D (G91) ----------g--------------------t-----c---------c---t---c--a------t--------a-c--t 2000

D (G242) ----------g--------------------t-----c---------c---t---c--a------t--------a-c--t 2000

H (JIC15) -------------------------------t-----------------------------------------t--g--t 2000

H (CAR11) -------------------------t-----t-----------------------------------------t--g--t 2000

I (G374) ----g--c-g---------------t-----g-------------------cg---------------------atg--t 2000

I (G375) ----g----g---------------t-----g-------------------cg---------------------atg--t 2000

I (JIC13) ----g--c-g---------------t--a--g-------------------cg-----a---------------atg--t 2000

A (G180) GTGGCAGGATGAGGATTGCTGTAAGGCTCTGCAATGTCCGGGAACAGGCGACTAGCTGAGTACTCAAAGTATGACAGTTT 2080

A (M34) --------------------------------------------t----------------------------------- 2080

A (G191) -------------------------------------------------------------------------------- 2080

A (KAR) -------------------------------------------------------------------------------- 2080

A (G263) -------------------------------------------------------------------------------- 2080

A (NAC1) -------------------------------------------------------------------------------- 2080

C (G169) -at------------c--g--a-t-tt-----------------g-----------------t----------------- 2080

B (KEN) ----t-------------g-------------------------g-----------------t--------gtt------ 2080

E (JIC10) -aa-tt--------gc-ag--a-g-tt-----------------g----------------------------------- 2080

E (G359) -aa-tc--------gc-ag--a-g-t------------------g----------------------------------- 2080

E (G363) -aa-tt--------gc-ag--a-g-t------------------g----------------------------------- 2080

F (G364) --t---------------g----g-a------g-----t-----g--------------------------a-------- 2080

G (G384) --t---------------g----g-a------------t-----g--------------------------a-------- 2080

G (G385) --t---------------g----g-a------------t-----g-----------------t--g-----a-------- 2080

G (G386) --t---------------g----g-a------------t-----g-----------------t--g-----a-------- 2080

G (G383) --t-g-------------g----g-a------------t-----g--------------------------a-------- 2080

D (G91) ------------------g----g-a------g-----t-----g-----------------t--------a-------- 2080

D (G242) ------------------g----g-a------g-----t-----g-----------------t--------g-------- 2080

H (JIC15) -a---------t---c--g--a-g-tt-----------------g--------------------------g-------- 2080

H (CAR11) -at--t-----c--gc--g--a-g-tt-----------------g--------------------------g-------- 2080

I (G374) ----attcg---------g----g-t------------t-----g----------------------------------- 2080

I (G375) ---ttttcg---------g----g--------------t-----g----------------------------------- 2080

I (JIC13) -----ttc----------g----g--------------t-----g----------------------------------- 2080

A (G180) TGTGGCCCAGTCGTAAGGCAGTGAAGTCTGCACTAGTGAAAGGTACTCTGCTCTGCTAGTGGCATGTGTCATGATTTCCT 2160

A (M34) ---------------------------------------------------------------------------c---- 2160

A (G191) -----------------------------------------------------g---------------------c---- 2160

A (KAR) ---------------------------------------------------------------------------c---- 2160

A (G263) ----c----------------------------------------------------------------------c---- 2160

A (NAC1) ---------------------------------------------------------------------------c---- 2160

C (G169) ---t--------a--c-----gctgc----a-----a------------t----------a--------------c--tc 2160

B (KEN) ---a--------a--c--g--gctgc----------a------------t-c--a-----a--------------c--tc 2160

E (JIC10) a--a--------a--c-----gctgc----a--c--a------------t----------a--------------g--tc 2160

E (G359) a--a--------a--c-----gctgc----a--c--a------------t----------a--------------c--tc 2160

E (G363) a--a--------a--c-----gctgc----a--c--a------------t----------a--------------c--tc 2160

F (G364) ---t--------------------------------a------------t-ct-------a--------------c--tc 2160

G (G384) c--t-----------------c--t-c---g--c--a------------t-ct----g-----------------c--tc 2160

G (G385) c--t-----------------c--t-c---g--c--a------------t-ct----g-----------------c--tc 2160

G (G386) c--t-----------------c--t-c---g--c--a------------t-ct----g-----------------c--tc 2160

G (G383) c--t--------------t--c--t-c---g-----a------------t-ct----g-----------------c--tc 2160

D (G91) ---t--------a--g-----------------c--a------------t-ct----g-----------------c--tc 2160

D (G242) ---t--------a--g-----------------c--a------------t-ct----g-----------------c--tc 2160

H (JIC15) ---t--------a--c-----gctgc----a--c--a------------t-c--------a-----a-----------t- 2160

H (CAR11) ---t--------a--c-----gctgc----a--c--a------------t-c--------a-----a-----------t- 2160

I (G374) ------------a--g-------c-c-------c-t------------ct----agat--t--g--c--------c--t- 2160

I (G375) ---------a--a--g-------c-c-------c-t------------ct----agag--t--g--c-----------t- 2160

I (JIC13) ------------a--g-------c-c-------c-t------------ct----agat--t--g--c-----------tc 2160

A (G180) TCATGACTTCATCCTTAGAAGGCTTCTTCTCAGTATT---CTTACCTGGAGCGACGAAGCTCTTCTTCCTTGGAATGTAA 2240

A (M34) -------c-------------------------a---ttc-----------ga--------------------t------ 2240

A (G191) -------c-------------------------a---ttc-----------ga--------------------t------ 2240

A (KAR) -------c-------------------------ag--ttc-----------ga--------------------t------ 2240

A (G263) -------c-------------------------a---ttc-----------ga--------------------t------ 2240

A (NAC1) -------c-------------------------a---ttc-----------gt--------------------t------ 2240

C (G169) ------------------t--------------a-c-ctc---------c-gact----ga------------------t 2240

B (KEN) ----------------g-t---t----------ctggctc-----------g---------------------g-----t 2240

E (JIC10) ----------------gct--------------a-c-atc---------t-g-ct----ga------------------t 2240

E (G359) ----------------gct--------------a-c-atc---------t-gact----ga------------------t 2240

E (G363) ----------------gct--------------a-c-atc---------t-gact----ga------------------t 2240

F (G364) ------t---------gct--------------ag--ttc-----------g-------ga------------t-----t 2240

G (G384) -------c--------gct--------------aggagtc-----------g---------------------g--a--t 2240

G (G385) -------c--------gct--------------aggagtc-----------g---------------------g-----t 2240

G (G386) -------c--------gct--------------aggagtc-----------g---------------------g--a--t 2240

G (G383) ----------------gct--------------aggagtc-----------g---------------------g-----t 2240

D (G91) ----------------gct--------------ag--ttc-----------gc------ga------------t-----t 2240

D (G242) ----------------gct----------c---ag--ttc-----------gc------ga------------t-----t 2240

H (JIC15) ----------------gct--------------ctggttc-----------g--------a------------g-----t 2240

H (CAR11) -------c--------gct--------------ctggttc-----------g-------ga--------g---t-----t 2240

I (G374) ------tc--------gct--------------agggttct--------c-g-ga----ga---------g--t-----t 2240

I (G375) ------tc--------gct--------------atggttct--------c-gtga----ga---------g--t-----t 2240

I (JIC13) -------c--------gct--------------atggttc---------t-g-ga----ga---------g--t-----t 2240

A (G180) GTACCTTTCTCCCACTTGTCCTTGGGATCCTTCAGTATGTACTCACGTACCTTGTCAGTGCTCTTGGCGCTCTGTATATT 2320

A (M34) -----------------------------------------------------------a-------------------- 2320

A (G191) --------------------------g--------------------------------a-------------------- 2320

A (KAR) -------------------------------------------------------------------------------- 2320

A (G263) -------------------------------------------------------------------------------- 2320

A (NAC1) -------------------------------------------------------------------------------- 2320

C (G169) -----------------------c-----------------g-----c-----------------------------g-- 2320

B (KEN) --------------------------g--------------------c-----------------------------g-- 2320

E (JIC10) --------------------------g--------------g-----c-----------------------------g-- 2320

E (G359) --------------------------g--------------g-----c-----------------------------g-- 2320

E (G363) --------------------------g--------------g-----c-----------------------------g-- 2320

F (G364) --------------------------g--------------------c-----------------------------g-- 2320

G (G384) --g-----------------------g-t------------g-----c-------------------------------- 2320

G (G385) --g-----------------------g-t------------g-----c-------------------------------- 2320

G (G386) --g-----------------------g-t------------g-----c-------------------------------- 2320

G (G383) --g-----------------------g-t------------g-----c-----------------------t-----g-- 2320

D (G91) --------------------------g--------------g-----c-----------------------------g-- 2320

D (G242) --------------------------g--------------g-----c-----------------------------g-- 2320

H (JIC15) --------------------------g--------------g-----c--------g--------------------g-- 2320

H (CAR11) --------------------------g--------------g-----c-----------------------------g-- 2320

I (G374) -----------------------c-----------g-----ag-t--c--------g-----------------a--g-- 2320

I (G375) -----------------------c-----------g-----ag-t--c--------------------------a--g-- 2320

I (JIC13) -----------------c--t--c-----------g-----agat--c--------g-----------------a----- 2320

A (G180) CGGATGATACCGATCTATGTCAAAGTACCTCTCGTCACGAGTAGTGACAGGCTTGATGCACTGGAGAAGAGCATGGCAGT 2400

A (M34) ---------------------------------a---------------------------------------------- 2400

A (G191) -------------------------------------------------------------------------------- 2400

A (KAR) -------------------------------------------------------------------------------- 2400

A (G263) -------------------------------------------------------------------------------- 2400

A (NAC1) ---------------------------t---------------------------------------------------- 2400

C (G169) ------g------------------------------------------------------------------------- 2400

B (KEN) -cc---g---------------------------------------ca------------t--c--g--t---------- 2400

E (JIC10) g--g--g------------------------------------------------------------------------- 2400

E (G359) g-----g-----------------------------c------------------------------------------- 2400

E (G363) g-----g------------------------------------------------------------------------- 2400

F (G364) g-----g------------------------------------------------------------------------- 2400

G (G384) g-----g--------g--------------------------------g--t--------------c------------- 2400

G (G385) g-----g--------g--------------------------------g--t--------------c------------- 2400

G (G386) g-----g--------g--------------------------------g--t--------------c------------- 2400

G (G383) ------g--------g--------------------------------g--t--------------c------------- 2400

D (G91) g-----g--------------------------a--t-t---t-----t-----------t--c--g------------- 2400

D (G242) g-----g--------------------------a--t-tt--t-----t-----------t--c--g------------- 2400

H (JIC15) g-----g--------g-----------------a--c-t---t-----t-----------t--c--g------------- 2400

H (CAR11) g-----g--------------------------a--c-t---t-----t-----------t--c--g------------- 2400

I (G374) g--g--g--t-----------g-----g-g------g-tc--gc-t--t------g--gt---a--g------------- 2400

I (G375) g--g--g--t-----------g-----g-g------g-tc--gc-t--t------g--gt---a--g------------- 2400

I (JIC13) g--g--g--t-----------g-----g-g------g-tt--gc-t--t--tc--g--gtt--a--g------------- 2400

A (G180) GCCATGTTCCGTCTTGGTGAGTCTCTCTCACTACAAGGATGTATGCTGGCTCAAAGTCCTTAGTGAGCCTGAAGAGATGC 2480

A (M34) ----------a-----------------------c--------------------------------------------- 2480

A (G191) ----------------------------------c-----------------g--------------------------- 2480

A (KAR) ----------------------------------c--------------------------------------------- 2480

A (G263) ----------------------------------c--------------------------------------------- 2480

A (NAC1) ----------a-----------------------c--------------------------------------------- 2480

C (G169) -------c------gc---t--------g--c--t--t-----a------------------------------------ 2480

B (KEN) -------c------gt---t-----c--g-----t-a----------------t-t---c-------t------------ 2480

E (JIC10) --------------gt---t--------g-----t--t-----g---------t-------------------------- 2480

E (G359) --------------gt---t--------g-----t--t-----g---------t-------------------------- 2480

E (G363) --------------gt---t--------g--a--t--t-----g---------t-------------------------- 2480

F (G364) --------------at---t--------g-----t--t-----g---------t-------------------------- 2480

G (G384) -------c------aa---t-------gg-----c-a------------t--gt-------------------------- 2480

G (G385) -------c------aa---t-------gg-----t--------------t--gt----t---c----------------- 2480

G (G386) -------c------aa---t-------gg-----t-a------------t--gt-------------------------- 2480

G (G383) -------c------aa---t-------gg-----t-a------------t--gt--------c----------------- 2480

D (G91) -------c--a---gt---t--------a-----c--t-----g-----t-----------------------c------ 2480

D (G242) ----------a---gt---t--------a-----c--t-----g-----t---------c-------------c------ 2480

H (JIC15) -------c--a--c-----t--------g-----c--t-----g-----t------------------------------ 2480

H (CAR11) -------c-----c-----t--------g-----t--t-----------t--g------c-------------------- 2480

I (G374) -------c--a---at---t--------g-----t--------g-----g---t-------g-----t-----c------ 2480

I (G375) -------c--a---at---t--------g-----t--------g-----g---t-------g-----t--t--------- 2480

I (JIC13) -------c--a---at---t--------g-----t-a------g-----g-----------g-----t------------ 2480

A (G180) TCACCGATGAACTCTGGTTCTAGTGGACACTTGCTGTATGTGAGGAAGGTGTTCGCGTTCCGGTGCCTGAAGGACCGGAC 2560

A (M34) --------------a--------------------------------------t--------------a----------- 2560

A (G191) -------------g------------------------------------------------------------------ 2560

A (KAR) --------------a----------------------------------------------------------------- 2560

A (G263) --------------a----------------------------------------------------------------- 2560

A (NAC1) -------------------------------------------------------------------------------- 2560

C (G169) --------------------------------------------------------------------a---ct------ 2560

B (KEN) ------------------------------------------------------------------------c-a----- 2560

E (JIC10) --------------c--------a-----------------------------aa-------------a---ct-----t 2560

E (G359) --------------c--------a-----------------------------aa-------------a---ct-----t 2560

E (G363) --------------c--------------t-----------------------aa-------------a---ct-----t 2560

F (G364) -----------------------a-----t-----------------------aa-a-----------a---ct------ 2560

G (G384) -----------------------a---a-------------------------aa-------------a---ct--c--t 2560

G (G385) -----------------------a-----------------------------aa-------------a---ct-----t 2560

G (G386) --------------c--------a-----------------------------aa-------------a---ct-----t 2560

G (G383) -----------------------a-----------------------------aa-------------a---ct-----t 2560

D (G91) -----------------------------------------c-----------t------------------c-a----- 2560

D (G242) --------------------------------------g--c-----------t------------------c-a----- 2560

H (JIC15) --------------------------------------g--c-----------t------------------c-g----- 2560

H (CAR11) -----------------------------tc-------g--c-----------t------------------c-g----- 2560

I (G374) ------------------------------------------------------a-------a----ga-----t----- 2560

I (G375) -----------------------------tc-----------------------a-------a----ga-----t----- 2560

I (JIC13) ------------------------------------------------------a-------a----ga-----t----- 2560

Replicationassociated protein start codon

A (G180) GCTGTGCCTGCCATCAGAGGTGATTGACAGAGA------------GGTCGACATTAGCGCAGGAGGTTG--GGCA--GTC 2640

A (M34) ------t----------------------------------------------------------------------a-- 2640

A (G191) ------------------------a----------------------------------------------------a-- 2640

A (KAR) ---------------t--------a----------------------------------------------------a-- 2640

A (G263) ------------------------a----------------------------------------------------a-- 2640

A (NAC1) ------------------------------------------------------------------c----------a-- 2640

C (G169) ------------------------------------------------------------------c----------a-- 2640

B (KEN) ------------c---------------------gatgatcctac---------------------c--------g-a-- 2640

E (JIC10) cg----------tgtg-gt--c-ca-tg--g--ggttgatccttc---------------ta-------------a---- 2640

E (G359) cg----------tgtg-gt--c-ca-tg--g--ggttgatccttc---------------ta-------------a---- 2640

E (G363) cg----------tgtg-gt--t-ca-tg--g--ggttgatccttc---------------ta-c-----------a---- 2640

F (G364) tg----------tgtg-g---c-ca-tg--g--ggttgatccttc---------------ta-------------a---- 2640

G (G384) cg-----------gtg-ct----cattg--g--ggttgatccttc---------------ta-------------gat-- 2640

G (G385) cg-----------gtg-ct----cattg--g--ggttgatccttc---------------ta-------------gat-- 2640

G (G386) cg-----------gtg-ct----ca-tg--g--ggttgatccttc---------------ta-------------gat-- 2640

G (G383) cg-----------gtg-ct----cattg--g--ggttgatccttc---------------ta-------------gat-- 2640

D (G91) -----------tc------c-------g-a----------cctac---g-----------ta-------------g---- 2640

D (G242) -----------tc------c-------g-a----------ccaac---g-----------ta-------------g---- 2640

H (JIC15) ---------a--g-----a----ca--g-------------ctt----------------ta-------------a---- 2640

H (CAR11) ---------a--g-----a----ca--g-------------ctg----------------ta-------------a---- 2640

I (G374) -g-------t---gtctg--a-ga-ccg-cg-------------ta--ttc----t-----a-------at----g--a- 2640

I (G375) -g-------t---gtctg--a-ga-tcg-cg-------------ta---tc----t-----a-------at----g--a- 2640

I (JIC13) -g-------a---gtctg--a-gaatcg-cg-------------ta---tc----t-----a-------at----g--a- 2640

A (G180) TCAGGCTC-TTCTCAGCTCTACCCCTAGACA--GCTGCGAAATAATCCGCCCACCGCC-CGTG-CCTTTTATAG-CTGCT 2720


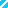

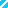


Iteron[3]

TATA box[5,7]

A (M34) ----------c----------t---------------------------------c------------------------ 2720


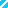


A (G191) ----------c----------t---------------------------------------------------------- 2720

A (KAR) ----------c----------t---------------------------------c------------------------ 2720

A (G263) ----------c----------t---------------------------------c------------------------ 2720

A (NAC1) ----------c----------t---------------------------------c------------------------ 2720

C (G169) ----------c----a-----t---------------------------------c--tgc-------------tg--t- 2720

B (KEN) ----a-----caa-t--------a-------------------------------c------cc----------tg-tt- 2720

E (JIC10) ----------c----a-----t---------------------------------c------cc-----------t---- 2720

E (G359) ----------c----a---------------------------------------c------cc-----------t---- 2720

E (G363) --------c-c----a-----t---------------------------------c---a--ct---------a-t-a-- 2720

F (G364) --------c-c----a---------------------------------------c------ccg----------t---- 2720

G (G384) -------gc-c----a------t--------cc----------------------c--------g----------t--a- 2720

G (G385) -------gc-c----a------t--------cc----------------------c--------g----------t---- 2720

G (G386) -------gc-c----a------t--------cc----------------------c--------g----------t---- 2720

G (G383) -------gc-c----a------t--------cc----------------------c--------g----------t---- 2720

D (G91) --------c-c----a-----t---------------------------------c---a---c-----------t--t- 2720

D (G242) --------c-c----a-----t---------------------------------c---a---c-----------t--t- 2720

H (JIC15) --------c-c----a-----t-------------------------t-------c--g----c-----------t--t- 2720

H (CAR11) --------c------a-----t---------------------------------c-------c-----------t---- 2720

I (G374) ----a-----c-ca-a-----t-----------------------------ac-a---ga---t----------c-g-a- 2720

I (G375) ----a-----c-ca-a-----t-----------------------------ac-a---ga---t-t--------c-g-a- 2720

I (JIC13) ----a----cc-ca-a-----t-----------------------------ac-a---ga------------------gg 2720

GC-box (rightward promoter element)[10]

Virion strand origin of replication[1]

Inverted repeat[2]


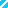

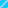


A (G180) T-GGTGGGCTGGGCCGGCCGGCCC-ATGGGG-TGTGAGCAGCATAATATT 2769


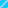


A (M34) -------------------------------------------------- 2769

A (G191) -------------------------------------------------- 2769

A (KAR) -------------------------------------------------- 2769

A (G263) -------------------------------------------------- 2769

A (NAC1) -------------------------------------------------- 2769

C (G169) ------------------------a------g------------------ 2769


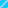


B (KEN) -------------------------------------------------- 2769

E (JIC10) -------------------------------------------------- 2769

E (G359) ------------------a------------------------------- 2769

E (G363) -------------------------------------------------- 2769

F (G364) -------------------------------------------------- 2769

G (G384) -aa-------------a--------------------------------- 2769

G (G385) -aa----------------------------------------------- 2769

G (G386) -aa----------------------------------------------- 2769

G (G383) -aa----------------------------------------------- 2769

D (G91) -------------------------------------------------- 2769

D (G242) -------------------------------------------------- 2769

H (JIC15) -------------------------------------------------- 2769

H (CAR11) --------t----------------------------------------- 2769

I (G374) -a---------------------------a-------------------- 2769

I (G375) -a---------------------------a-------------------- 2769

I (JIC13) -aa--------------------------a-------------------- 2769
