# Supplementary material for: Comparative analysis of Panicum streak virus and Maize streak virus diversity, recombination patterns and phylogeography
Source: Virol J. 2009 Nov 10;6:194. doi: 10.1186/1743-422X-6-194 (PMC2777162; doi:10.1186/1743-422X-6-194)
Supplement: Additional file 3 — Annotated predicted movement protein amino acid sequence alignments. Annotated predicted movement protein amino acid sequence alignments of 23 PanSV isolates. The hydrophobic, potentially membrane spanning internal domain of the sequences is highlighted. [1] Wright et al.1997. Plant J. 12:1285. [file 1743-422X-6-194-S3.doc]

PanSV-A [ZM-Nya-g180-2007] MDASSTTP-FPFPQPPRVPSAAPVAGGLPWSRVGEIVIFTFVSVLGLYLLWLWVLRDCILLLKAQRGRSTEELIFGPGERPAVACADGSRPVPDPSPSCPPGPRPFVV

Hydrophobic potential trans-membrane domain[1]

PanSV-A [ZA-Bak-M34-2005] MDASSSTP-FPFPQPPRVPSAAPVAGGLPWSRVGEIVIFTFVSVLGLYLLWLWVLKDCILLLKAQRGRSTEELIFGPGERPAVASADGSRPVPDPSPSCPPGPRPFVV

PanSV-A [ZA-For-g191-2007] MDASSTTP-FPFPQPPRVPSAAPVAGGLPWSRVGEIVIFTFVSVLGLYLLWLWVLKDCILLLKAQRGRSTEELIFGPGERPAVASADGSRSVPDPSPSCPPGPRPFVV

PanSV-A [ZA-Kar-1994] MDASSTTP-FPFPQPPRVPSAAPVAGALPWSRVGEIVIFTFVSVLGLYLLWLWVLKDCILLLKAQRGRSTEELIFGPGERPAVASADGSRSVPDPSPSCPPGPRPFVV

PanSV-A [ZA-Ill-g263-2008] MDASSTTP-FPFPQPPRVPSAAPVAGGLPWSRVGEIVIFTFVSVLGLYLLWLWVLKDCILLLKAQRGRSTEELIFGPGERPAVASADGSRSVPDPSPSCPPGPRPFVV

PanSV-A [MZ-Nac1-2009] MYASSTTP-FPFPQPPRVPSAAPVAGGLPWSRVGEIVIFTFVSVLGLYLLWLWVLKDCILLLKAQRGRSTEELIFGPGERPAVASADGSRSVPDPSPSCPPGPRPFVV

PanSV-C [ZM-NGur-g169-2006] MDASSTTP-FPFPQPPRVPSAAPSAGGLPWSRVGEIVIFTFVSVLGLYLLWLWVLKDCILLLKAQRGRSTEELIFGPGERPAVASADGSRPVPDPSPSCPPGPRPFVV

PanSV-B [KE-Ken-1991] MDASSQYSALPYPQPPRVPSAAPSAGRLPWSRVGEIVIFTFVSVLALYLLWLWVLKDCILLLKAQRGRSTEELIFGPGERPPVASADGSRPVPDPSPPVRRDLDLSRV

PanSV-E [KE-Jic10-PKPM-1997]MDASNQYSALPYPQPPRVPSAAPSAGGLPWSRVGEIAIFTFVSVLALYLLWLWVLRDLILLLKAQRGRSTEELIFGPGERPAVAAADGSRPVSDPSPSCPPGPRPFVV

PanSV-E [KE-Nye5-g359-2008] MDASSQYSALPYPQPPRVPSAAPSAGGLPWSRVGEIAIFTFVSVLALYLLWLWVLRDLILLLKAQRGVSTEELIFGPGERPAVAAADGSRPVSDPSRPVHRDLDLSWV

PanSV-E [KE-Nye4-g363-2008] MDASSQYSALPYPQPPRVPSAAPSAGGLPWSRVGEIAIFTFVSVLALYLLWLWVLRDLILLLKAQRGRSTEELIFGPGERPAVAAADGSRPVSDPSPSCPPGPRPFVV

PanSV-F [KE-Nye2-g364-2008] MDASSQYSALPYPQPPRVPSAAPSAGGLPWSRVGEIVIFTFVSVLALYLLWLWVLKDCILLLKAQRGRSTEELIFGPGERPAVAAADGSRPVPDPSPSCPPGPRPFTV

PanSV-G [YT-Ben-g384-2008] MDAQSSYSSLPYPSAPRVPSAAPSAGGLPWSRVGEIVIFTFVSVLALYLLWLWVLKDCILLLKAQRGRSTEELTFGPGESSAVVPADGSRPVADPSPSCPPGPRPFVV

PanSV-G [YT-Coc-g385-2008] MDAQSSYSSFPYPSAPRVPSAAPSAGGLPWSRVGEIVIFTFVSMLALYLLWLWVLKDCILLLKAQRGRSTEELTFGPGESSAVVPADGSRPVADPSPSCPPGPRPFVV

PanSV-G [YT-Tsa-g386-2008] MDAQSSYSSLPYPSAPRVPSAAPSAGGLPWSRVGEIVIFTFVSVLALYLLWLWVLKDCILLLKAQRGRSTEELTFGPGESSAVVPADGSRPVADPSPSCPPGPRPFVV

PanSV-G [YT-Com-g383-2008] MDAQSSYSSLPYPSAPRVPSAAPSAGGLPWSRVGETVIFTFVSVLALYLLWLWVLKDCILLLKAQRGRSTEELTFGPGESSVVVPADGSRPVADPSPSCPPGPRPFVV

PanSV-D [NG-Ifo-g91-2006] MEASSQYSSLPYPQPPRVPAAAPSAGGLPWSRVGEIAIFTFVAVLALYLLWLWVLRDLILLVKAQRGRSTEELIFGPGERPAVAPADGSRPVPDPSPSCPPGPRPFVA

PanSV-D [NG-Ola-g242-2007] MEASSQYSSLPYPQPPRVPAAAPSAGGLPWSRVGEIAIFTFVAVLALYLLWLWVLRDLILLVKAQRGRSTEELIFGPGERPAVAPADGSRPVPDPSPSCPPGPRPFVA

PanSV-H [NG-Jic15-PNP-1997] MEASSQYSSLPYPQPPRVPSAAPSAGGLPWSRVGEIAIFTFVAVLALYLLWLWVLRDLILLVKAQRGRSTEELIFGPGERPPVAPADGSRPLPDPSPSCPPGPRPFVA

PanSV-H [CF-Bai2-Car11-2008]MEATSQYSSLPYPQPPRVPSAAPSAGGLPWSRVGEIAIFTFVAVLALYLLWLWVLRDLILLVKAQRGRSTEELIFGPGERPPVAPADGPRPLPDPSPSCPPGPRPFVA

PanSV-I [KE-Nra1-g374-2008] MEASGQYSSQPYPVSPRVPSAAPSAGGLPWSRVGEIAIFTFVAVLALYLLWLWVLRDLILLLKAQRGRSTEELIFGPGERPPVASADGSRPVPVPSPSCPPVPSPFLG

PanSV-I [KE-Nra2-g375-2008] MEASGQYSSQPYPVSPRVPSAAPSAGGLPWSRVGEIAIFTFVAVLALYLLWLWVLRDLILLLKAQRGRSTEELIFGPGERPPVASADGSRPVPVPSPSCPPVPSPFLG

PanSV-I [KE-Jic13-PKPB-1997]MEASGQYSSQPYPVSPRVPSAAPSAGGLPWSRVGEIAIFTFVAVLALYLLWLWVLRDLILLVKAQRGRSTEELIFGPGERPPVASADGSRPVPVPSPSCPPVPSPFVG
