# Supplementary material for: Comparative analysis of Panicum streak virus and Maize streak virus diversity, recombination patterns and phylogeography
Source: Virol J. 2009 Nov 10;6:194. doi: 10.1186/1743-422X-6-194 (PMC2777162; doi:10.1186/1743-422X-6-194)
Supplement: Additional file 4 — Annotated predicted coat protein amino acid sequence alignments. Annotated predicted coat protein amino acid sequence alignments of 23 PanSV isolates. The potential nuclear localization signal and DNA binding domains (inferred by analogy with those determined for MSV) are highlighted on the sequence. [1] Liu et al. 1999. Mol. Plant Microbe Interact. 12:894; [2] Liu et al. 1997. J. Gen. Virol. 78:1265. [file 1743-422X-6-194-S4.doc]

PanSV-A [ZM-Nya-g180-2007] MSGALKRKRSDEVAWSRRKPVKKQAR--VPPARAGPSVRRGLPALQIQTLIAAGDTMITVPSGGICSLIGTYARGSDEGNRHTNETLTYKVALDYHFVATAAACKYSSIGVGVMWLVYDAQPTGNSPEVKDIFPHSDTLSAFPYTWKVGREVCHRFVVKRRWCFTMETNGRIGSDVPPANTAWPPCKKDIYFHKFCTGLGVKTEWKNVTDGKVGAIKKGALYIVIAPGNGLEFTVHGQCRLYFKSVGNQ

Potential nuclear localization signal (MSV)[1]

DNA binding domain[2]

PanSV-A [ZA-Bak-M34-2005] MSGALKRKRSDEVAWSRRKPVKKQAR--VPPPRAGPSVRRGLPALQIQTLIAAGDTMITVPSGGICSLIGTYARGSDEGNRHTNETLTYKVALDYHFVATAAACKYSSIGVGVMWLVYDAQPTGNSPEVKDIFPHSDTLSAFPYTWKVGREVCHRFVVKRRWCFTMETNGRIGSDIPPANTAWPPCKKDLYFHKFCTGLGVKTEWKNVTDGKVGAIKKGALYIVIAPGNGLEFTVHGQCRLYFKSVGNQ

PanSV-A [ZA-For-g191-2007] MSGALKRKRSDEVAWSRRKPVKKQAR--VPPPRAGPSVRRGLPALQIQTLIAAGDTMITVPSGGICSLIGTYARGSDEGNRHTNETLTYKVALDYHFVATAAACKYSSIGVGVMWLVYDAQPTGNSPEVKDIFPHSDTLSAFPYTWKVGREVCHRFVVKRRWCFTMETNGRIGSDIPPANTAWPPCKKDLYFHKFCTGLGVKTEWKNVTDGKVGAIKKGALYIVIAPGNGLEFTVHGQCRLYFKSVGNQ

PanSV-A [ZA-Kar-1994] MSGALKRKRSDEVAWSRRKPVKKQDTG-FPLPRAGPSVRRGLPALQIQTLTAAGDTMITVPSGGICSLIGTYARGSDEGNRHTNETLTYKVALDYHFVATAAACKYSSIGVGVMWLVYDAQPTGNSPEVKDIFPHSDTLSAFPYTWKVGREVCHRFVVKRRWCFTMETNGRIGSDVPPANTAWPPCKKDIYFHKFCTGLGVKTEWKNVTDGKVGAIKKGALYIVIAPGNGLEFTVHGQCRLYFKSVGNQ

PanSV-A [ZA-Ill-g263-2008] MSGALKRKRSDEVAWSRRKPVKKQAR--VPPPRAGPSVRRGLPALQIQTLTAAGDTMITVPSGGICSLIGTYARGSDEGNRHTNETLTYKVALDYHFVATAAACKYSSIGVGVMWLVYDAQPTGNSPEVKDIFPHSDTLSAFPYTWKVGREVCHRFVVKRRWCFTMETNGRIGSDVPPANTAWPPCKKDIYFHKFCTGLGVKTEWKNVTDGKVGAIKKGALYIVIAPGNGLEFTVHGQCRLYFKSVGNQ

PanSV-A [MZ-Nac1-2009] MSGALKRKRSDEVAWSRRKPVKKQAR--VPPPRAGPSVRRGLPALQIQTLTAAGDTMITVPSGGICSLIGTYARGSDEGNRHTNETLTYKVALDYHFVATAAACRYSSIGVGVMWLVYDAQPTGNSPEVKDIFPHSDTLSAFPYTWKVGREVCHRFVVKRRWCFTMETNGRIGSDVPPANTAWPPCKKDLYFHKFCTGLGVKTEWKNVTDGKVGAIKKGALYIVIAPGNGLEFTVHGQCRLYFKSVGNQ

PanSV-C [ZM-NGur-g169-2006] MSGALKRKRSDEVAWSRRKPVKKPARRPPPP-RAGPSVRRGLPALQIQTLVAAGDTMITVPSGGICSLIGTYARGSGEGERHTNETLTYKVALDYHFVATAAACKYSSIGVGVCWLVYDAQPTGTAPTVQDIFPHPTTLAAFPYTWKVGREVCHRFVVKRRWCFTMETNGRIGSDVPPSNTAWPPCKKDIYFHKFSTGLGVKTEWKNVTDGKDGAIKKGGLYIVIAPGNGLEFTCHGQCRLYFKSVGNQ

PanSV-B [KE-Ken-1991] MSGALKRKRSDEVAWSRRRPVKKPVRRAPPP-RAGPSVRRGLPSLQIQTLVAAGDTMITVPSGGICSLIGTYARGSGEGERHTNETLTYKVALDYHFVATAAACKYSSIGIGVCWLVYDAQPTGTAPTVQDIFPHPATLSAFPYTWKVGREVCHRFVVKRRWCFTMETNGRIGSDTPPSNVAWPPCKKDIYFHKFCTGLGVKTEWKNVTDGKDGAIKKGGFYIVIAPGN-VEFTCHGQCRLYFKSVGNQ

PanSV-E [KE-Jic10-PKPM-1997]MSGALKRKRSDEVAWSRRKPVKKPARRPPPP-RAAPSVRRGLPSLQIQTLVAAGDTMITVPSGGICSLIGTYARGSGEGERHTNETLTYKVALDYHFVATAAACKYSSIGIGVCWLVYDAQPTGTAPTVQDIFPHPATLSAFPYTWKVGREVCHRFVVKRRWCFTMETNGRIGSDTPPSNVAWPPCKKDIYFHKFCTGLGVKTEWKNVTDGKDGAIKKGGFYIVIAPGNGLEFTCHGQCRLYFKSVGNQ

PanSV-E [KE-Nye5-g359-2008] MSGALKRKRSDEVAWSRRKPVKKPARRPPPP-RAAPSVRRGLPSLQIQTLVAAGDTMITVPSGGICSLIGTYARGSGEGERHTNETLTYKVALDYHFVATAAACKYSSIGIGVCWLVYDAQPTGTAPTVQDIFPHPATLSAFPYTWKVGREVCHRFVVKRRWCFTMETNGRIGSDTPPSNVAWPPCKKDIYFHKFCTGLGVKTEWKNVTDGKDGAIKKGGFYIVIAPGNGLEFTCHGQCRLYFKSVGNQ

PanSV-E [KE-Nye4-g363-2008] MSGALKRKRSDEVAWSRRKPVKKPARRPPPP-RAAPSVRRGLPSLQIQTLVAAGDTMITVPSGGICSLIGTYARGSGEGERHTNETLTYKVALDYHFVATAAACKYSSIGIGVCWLVYDAQPTGTAPTVQDIFPHPATLSAFPYTWKVGREVCHRFVVKRRWCFTMETNGRIGSDTPPSNVAWPPCKKDIYFHKFCTGLGVKTEWKNVTDGKDGAIKKGGFYIVIAPGNGLEFTCHGQCRLYFKSVGNQ

PanSV-F [KE-Nye2-g364-2008] MSGAMKRKRSDEVAWSRRRPVKKPARRPPPP-RAGPSVRRGLPALQIQTLVAAGDTMITVPSGGICSLIGTYARGSGEGERHTNETLTYKVALDYHFVASSAACKYSSIGVGVCWLVYDAQPTGTAPTVQDIFPHPATLSAFPYTWKVGREVCHRFVVKRRWCFTMETNGRIGSDTPPSNVAWPPCKKDIYFHKFCTGLGVKTEWKNVTDGKDGAIKKGGLYIVIAPGNGLEFTVHGQCRLYFKSVGNQ

PanSV-G [YT-Ben-g384-2008] MSGALKRKRSDEVAWSRRKPVKKPARRPPPPPRAGSSSKRTLPALQIQTLVAAGDTMITVPSGGICSLIGTYARGSGEGERHTNETLTYKVALDYHFVATAAACKYSSIGIGVCWLVYDAQPTGTAPTVQDIFPHPDTLSAFPYTWKVGREVCHRFVVKRRWCFTMSTNGRIGSDVPPSNTAWPPCKKDLYFHKFCTGLGVKTEWKNVTDGKDGAIKKGGLYIVIAPGNGLEFTVHGQCRLYFKSVGNQ

PanSV-G [YT-Coc-g385-2008] MSGALKRKRSDEVAWSRRKPVKKPARRPPPPARAGSSSKRTLPALQIQTLVAAGDTMITVPSGGICSLIGTYGRGSGEGERHTNETLTYKVALDYHFVATAAACKYSSIGIGVCWLVYDAQPTGTAPTVQDIFPHPDTLSAFPYTWKVGREVCHRFVVKRRWCFTMSTNGRIGSDVPPSNTAWPPCKKDLYFHKFCTGLGVKTEWKNVTDGKDGAIKKGGLYIVIAPGNGLEFTVHGQCRLYFKSVGNQ

PanSV-G [YT-Tsa-g386-2008] MSGALKRKRSDEVAWSRRKPVKKPARRPPPPPRAGSSSKRTLPALQIQTLVAAGDTMITVPSGGICSLIGTYARGSGEGERHTNETLTYKVALDYHFVATAAACKYSSIGIGVCWLVYDAQPTGTAPTVQDIFPHPDTLSAFPYTWKVGREVCHRFVVKRRWCFTMSTNGRIGSDVPPSNTAWPPCKKDLYFHKFCTGLGVKTEWKNVTDGKDGAIKKGGLYIVIAPGNGLEFTVHGQCRLYFKSVGNQ

PanSV-G [YT-Com-g383-2008] MSGALKRKRSDEVAWSRRKPVKKPARRPPPPPRAGSSSKRTLPALQIQTLVAAGDTMITVPSGGICSLIGTYARGSGEGERHTNETLTYKVALDYHFVATAAACKYSSIGIGVCWLVYDAQPTGTAPTVQDIFPHPDTLSAFPYTWKVGREVCHRFVVKRRWCFTMSTNGRIGSDVPPSNTAWPPCKKDLYFHKFCTGLGVKTEWKNVTDGKDGAIKKGGLYIVIAPGNGLEFTVHGQCRLYFKSVGNQ

PanSV-D [NG-Ifo-g91-2006] MSGALKRKRSDEVAWSRRKPAKKPARQ-PPP-RAGPSVRRGLPALQIQTLVAAGDTMITVPSGGICSLLGTYARGSDEGNRHTNETLTYKVALDYHFVATAAACKYSSIGVGVCWLVYDAQPSGNAPEVKDIFPHGDTLSAFPYTWKVGREVCHRFVVKRRWTFTMETDGRIGSDVPPANASWPPCKRSIYFHKFCTGLGVKTEWKNVTDGKVGAIKKGALYIVLAPGNGLEFTVHGQARLYFKSVGNQ

PanSV-D [NG-Ola-g242-2007] MSGALKRKRSDEVAWSRRKPAKKPARQ-PPP-RAGPSVRRGLPALQIQTLVAAGDTMITVPSGGICSLLGTYARGSDEGNRHTNETLTYKVALDYHFVATAAACKYSSIGVGVCWLVYDAQPSGNAPEVKDIFPHGDTLSAFPYTWKVGREVCHRFVVKRRWTFTMETDGRIGSDVPPANASWPPCKRSIYFHKFCTGLGVKTEWKNVTDGKVGAIKKGALYIVLAPGNGLEFTVHGQARLYFKSVGNQ

PanSV-H [NG-Jic15-PNP-1997] MSGALKRKRSDEVAWSRRKPAKKPARQ-PPP-RAGPSVRRGLPALQIQTLVAAGETMITVPSGGICSLLGTYARGSDEGNRHTNETLTYKVALDYHFVATAAACKYSSIGVGVCWLVYDAQPSGNAPAVKDIFPHGDTLSAFPYTWKVGREVCHRFVVKRRWTFTMETDGRIGSDVPPPDASWPPCKRSIYFHKFCTGLGVKTEWKNVTDGKVGAIKKGALYIILAPGNGLEFTVNGQARLYFKSVGNQ

PanSV-H [CF-Bai2-Car11-2008]MSGALKRKRSDEVAWSRRKPAKKPARQ-PPPPRAGPSVRRGLPALQIQTLVAAGETMITVPSGGICSLLGTYSRGSDEGNRHTNETLTYKVALDYHFVATAAACKYSSIGVGVCWLVYDAQPSGNAPAVKDIFPHGDTLSAFPYTWKVGREVCHRFVVKRRWTFTMETDGRIGSDVPPPDASWPPCKRSIYFHKFCTGLGVKTEWKNVTDGKVGAIKKGALYIILAPGNGLEFTVHGQARLYFKSVGNQ

PanSV-I [KE-Nra1-g374-2008] MAGALKRKRSDEVAWSRRKPAKKPAR--APPPRAGPSVRRGLPALQIQSLVAAGETMITVPNGGICSPIVTYSRGSDEGNRHTNETLTYKIALDYHFVLSSAACKYASRAVGVVWLVYDAQPTGNAPQVKDIFPHGDALSAFPYTWKVGREVCHRFVVKRRWTFTMESNGRIGSDTPPENAAWTPVKRSIYFHKFCTGLGVKTEWKNVTDGGVGAIKKGALYIVIAPGNGLDFTVHGQCRLYFKSVGNQ

PanSV-I [KE-Nra2-g375-2008] MAGALKRKRSDEVAWSRRKPAKKPAR--APPPRAGPSVRRGLPALQIQSLVAAGETMITVPNGGICSPIVTYSRGSDEGNRHTNETLTYKIALDYHFVLSSAACKYASRAVGVVWLVYDAQPTGNAPQVKDIFPHGDALSAFPYTWKVGREVCHRFVVKRRWTFTMESNGRIGSDTPPENAAWTPVKRSIYFHKFCTGLGVKTEWKNVTDGGVGAIKKGALYIVIAPGNGLDFTVHGQCRLYFKSVGNQ

PanSV-I [KE-Jic13-PKPB-1997]MAGALKRKRSDEVAWSRRKPAKKPAR--APPPRAGPSVRRGLPALQIQTLVAAGDTMITVANGGICSPLVTYSRGSDEGNRHTNETLTYKIALDYHFVLSSAACKHASRAVGVVWLVYDAQPTGNAPQVKDIFPHGDTLSAFPYTWKVGREVCHRFVVKRRWTFTMESNGRIGSDTPPANAAWTPVKRSIYFHKFCTGLGVKTEWKNVTDGGVGAIKKGALYIVIAPGNGLDFTAHGQCRLYFKSVGNQ
