# Supplementary material for: Comparative analysis of Panicum streak virus and Maize streak virus diversity, recombination patterns and phylogeography
Source: Virol J. 2009 Nov 10;6:194. doi: 10.1186/1743-422X-6-194 (PMC2777162; doi:10.1186/1743-422X-6-194)
Supplement: Additional file 5 — Annotated predicted replication-associated protein amino acid sequence alignments. Annotated predicted replication-associated protein amino acid sequence alignments of 23 PanSV isolates. Potential rolling-circle replication motifs and interaction domains inferred by analogy with MSV and Wheat dwarf virus are highlighted. [1] Koonin & Ilyina. 1992. J Gen Virol, 73:2763; [2] Horvath et al. 1998. Plant Mol. Biol. 38:699; [3] Xie et al. 1995. EMBO J. 14:4073; [4] Gorbalenya & Koonin. 1989. Nucl. Acids Res. 17:8413. [file 1743-422X-6-194-S5.doc]

PanSV-A [ZM-Nya-g180-2007] MST----SLSITSDGRHSVRSFRHRNANTFLTYSKCPLEPEFIGEHLFRLTKDFEPAYILVVRETHQDGTWHCHALLQCIKPVTTRDERYFDIDRYHPNIQSAKSTDKVREYILKDPKDKWEKGTYIPRKKSFVAPGK-NTEKKPSKDEVMKEIMTHATSRAEYLSLVQTSLPYDWATKLSYFEYSASRLFPDIAEPYSNPHPATDPDLLCNETLQDWLEPNIYQITPGARKRSLYIVGPTRTGKTSWARSLGRHNYWQNNIDWSSYDEEAVYNVVDDIPFKFCPCWKQLVGCQKDYIVNPKYGKRRKVASKSIPTIILANEDEDWLKDMTPAQYDYFYANCEIYVMQAGEKWFTPA

Oligomerisation domain[2]

Non-functional retinoblastoma binding protein binding motif[3]

dNTP binding motif[4]

*myb*-like transactivation domain[2]

PanSV-A [ZA-Bak-M34-2005] MST----SLSITSDGRHSVRSFRHRNANTFLTYSKCPLEPEFIGEHLFRLTKDFEPAYILVVRETHQDGTWHCHALLQCIKPVTTRDERYFDIDRYHPNIQSAKSTDKVREYILKDPKDKWEKGTYIPRKKSFVPPGKENSEKKPSKDEVMKEIMTHATSRAEYLSLVQTSLPYDWATKLSYFEYSASRLFPDIAEPYSNPHPATDPDLLCNETLQDWLEPNIYQITPGARKRSLYIVGPTRTGKTSWARSLGRHNYWQNNIDWSSYDEEAVYNVVDDIPFKFCPCWKQLVGCQKDYIVNPKYGKRRKVASKSIPTIILANEDEDWLKDMTPAQYDYFYANCEIYVMQAGEKWFTPA

PanSV-A [ZA-For-g191-2007] MST----SLSITSDGRHSVRSFRHRNANTFLTYSKCPLEPQFIGEHLFRLTKDFEPAYILVVRETHQDGTWHCHALLQCIKPVTTRDERYFDIDRYHPNIQSAKSTDKVREYILKDPKDKWEKGTYIPRKKSFVPPGKENSEKKPSKDEVMKEIMTHATSRAEYLSLVQTSLPYDWATKLSYFEYSASRLFPDIAEPYSNPHPATDPDLLCNETLQDWLEPNIYQITPGARKRSLYIVGPTRTGKTSWARSLGRHNYWQNNIDWSSYDEEAVYNVVDDIPFKFCPCWKQLVGCQKDYIVNPKYGKRRKVASKSIPTIILANEDEDWLKDMTPAQYDYFYANCEIYVMQAGEKWFTPA

PanSV-A [ZA-Kar-1994] MST----SLSITSDGRHSVRSFRHRNANTFLTYSKCPLEPEFIGEHLFRLTKDFEPAYILVVRETHQDGTWHCHALLQCIKPVTTRDERYFDIDRYHPNIQSAKSTDKVREYILKDPKDKWEKGTYIPRKKSFVPPGKENSEKKPSKDEVMKEIMTHATSRAEYLSLVQTSLPYDWATKLSYFEYSASRLFPDIAEPYSNPHPATDPDLLCNETLQDWLEPNIYQIIPGARKRSLYIVGPTRTGKTSWARSLGRHNYWQNNIDWSSYDEEAAYNVVDDIPFKFCPCWKQLVGCQKDYIVNPKYGKRRKVASKSIPTIILANEDEDWLKDMTPAQYDYFYANCEIYVMQAGEKWFTPA

PanSV-A [ZA-Ill-g263-2008] MST----SLSITSDGRHSVRSFRHRNANTFLTYSKCPLEPEFIGEHLFRLTKDFEPAYILVVRETHQDGTWHCHALLQCIKPVTTRDERYFDIDRYHPNIQSAKSTDKVREYILKDPKDKWEKGTYIPRKKSFVPPGKENSEKKPSKDEVMKEIMTHATSRAEYLSLVQTSLPYDWGTKLSYFEYSASRLFPDIAEPYSNPHPATDPDLLCNETLQDWLEPNIYQIIPGARKRSLYIVGPTRTGKTSWARSLGRHNYWQNNIDWSSYDEEAAYNVVDDIPFKFCPCWKQLVGCQKDYIVNPKYGKRRKVASKSIPTIILANEDEDWLKDMTPAQYDYFYANCEIYVMQAGEKWFTPA

PanSV-A [MZ-Nac1-2009] MST----SLSITSDGRHSVRSFRHRNANTFLTYSKCPLEPEFIGEHLFRLTKDFEPAYILVVRETHQDGTWHCHALLQCIKPVTTRDERYFDIDRYHPNIQSAKSTDKVREYILKDPKDKWEKGTYIPRKKSFVPPGKENSEKKPSKDEVMKEIMTHATSRAEYLSLVQTSLPYDWATKLSYFEYSASRLFPDIAEPYSNPHPATDPDLLCNETLQDWLEPNIYQITPGARKRSLYIVGPTRTGKTSWARSLGRHNYWQNNIDWSSYDEEAVYNVVDDIPFKFCPCWKQLVGCQKDYIVNPKYGKRRKVASKSIPTIILANEDEDWLKDMTPAQYDYFYANCEIYVMQAGEKWFTPA

PanSV-C [ZM-NGur-g169-2006] MST----SLSITSDGRHSVRSFRHRNANTFLTYSKCPLEPEFIGEHLFRLTKDFEPAYILVVRETHADGTWHCHALLQCIKPVTTRDERYFDIDRYHPNIQSAKSTDKVRDYILKDPKDKWEKGTYIPRKKSFSPPGKESSEKKPTKDEVMREIMTHATSREEYLSLVQSSLPYDWATKLSYFEYSASRLFPDIAETFTSPHPASDPDLLCNETLQDWLEPNIYQITPGARKTSLYIVGPTRTGKTSWARSLGRHNYWQNNIDWSSYDEEALYNVVDDIPFKFCPCWKQLVGCQTDYIVNPKYGKRRKVAKKSIPTIILANEDEDWLKELTPSQYDYFYANCEIYVMQAGEKWYTPA

PanSV-B [KE-Ken-1991] MSTVGSSSE-----GRHSVRCFRHRNANTFLTYSKCPLEPEFIGEHLFRLTREYEPAYILVVRETHTDGTWHCHALLQCIKPCTTRDERYFDIDRYHGNIQSAKSTDKVREYILKDPKDKWEKGTYIPRKKSFVPPGKEPAEKKPTKDEVMREIMTHATSREEYLSLVQSSLPYDWATKLNYFEYSASRLFPDIAEPYTNPHPTTEYDLHCNETIEDWLKPNIYQNAPGERKRSLYICGPTRTGKTSWARSLGRHNYWQNNIDWSSYDEEAQYNVVDDIPFKFCPCRKRLVGCQKDYIVNPKYGKRRKVASKSIPTIILANEDEDWLKDMTPAHVEYFEANCDQYILLPGEKFYKTGEAGGSI

PanSV-E [KE-Jic10-PKPM-1997]MSTEGSTSLTVTPTGRHTIRSFRHRNVNTFLTYSKCPLEPEFIGEHLFRLTKDYEPAYILVVRETHTDGTWHCHALLQCIKPVTTRDERYFDIDRYHPNIQSAKSTDKVRDYILKDPKDKWEKGTYIPRKKSFSPPGKDSSEKKPSKDEVMRDIMTHATSREEYLSLVQSSLPYDWATKLSYFEYSASRLFPDIAETFTSPHPTSEPDLLCNETLQDWLEPNIYQITPGARKTSLYIVGPTRTGKTSWARSLGRHNYWQNNVDWSSYDEEALYNVIDDIPFKFCPCWKQLVGCQTDYIVNPKYGKRRKVAKKSIPTIILANEDEDWLKELTPAQYEYFYANCEIYVMQAGEKWYTPA

PanSV-E [KE-Nye5-g359-2008] MSTEGSTSLTVTPTGRHTIRSFRHRNVNTFLTYSKCPLEPEFIGEHLFRLTKDYEPAYILVVRETHTDGTWHCHALLQCIKPVTTRDERYFDIDRYHPNIQSAKSTDKVRDYILKDPKDKWEKGTYIPRKKSFSPPGKDSSEKKPSKDEVMREIMTHATSREEYLSLVQSSLPYDWATKLSYFEYSASRLFPDIAETFTSPHPTSDPDLLCNETLQDWLEPNIYQITPGARKTSLYIVGPTRTGKTSWARSLGRHNYWQNNVDWSSYDEEALYNVIDDIPFKFCPCWKQLVGCQTDYIVNPKYGKRRRFAKKGIPTIILANEDEDWLKELTPAQYEYFYANCEIYVMQAGEKWYTPA

PanSV-E [KE-Nye4-g363-2008] MSTEGSTSLTVTPTGRHTIRSFRHRNVNTFLTYSKCPLEPEFIGEHLFRLTKDYEPAYILVVRETHTDGTWHCHALLQCIKPVTTRDERYFDIDRYHPNIQSAKSTDKVRDYILKDPKDKWEKGTYIPRKKSFSPPGKDSSEKKPSKDEVMREIMTHATSREEYLSLVQSSLPYDWATKLSYFEYSASRLFPDIAETFTSPHPTSDPDLLCNETLQDWLEPNIYQITPGARKRSLYIVGPTRTGKTSWARSLGRHNYWQNNVDWSSYDEEALYNVIDDIPFKFCPCWKQLVGCQTDYIVNPKYGKRRKVAKKSIPTIILANEDEDWLKELTPAQYEYFYANCEIYVMQAGEKWYTPA

PanSV-F [KE-Nye2-g364-2008] MSTEGSTSLTVTPTGRHTVRSFRHRNVNTFLTYSKCPLEPEFIGEHLFRLTKDYEPAYILVVRETHIDGTWHCHALLQCIKPVTTRDERYFDIDRYHPNIQSAKSTDKVREYILKDPKDKWEKGTYIPRKKSFVPPGKENSEKKPSKDEIMREIMTHATSKEEYLSLVQTSLPYDWATKLSYFEYSASRLFPDIAESYTNPHPATELDLHCNETIRDWLEPNIYQNAPGTRKRSLYIVGPTRTGKTTWARSLGRHNYWQNNVDWSSYDEEAQYNVVDDIPFKFCPCWKQLVGCQTDYIVNPKYGKRRKVAKKSIPTIILANEDEDWLKDMTPAQLSYFEANCDQYTLLSGEKFFQTE

PanSV-G [YT-Ben-g384-2008] MSTEGSTSLNVTATGRHTIGSFRHRNVNTFLTYSKFPLEPEFIGEHLFRLTKDYEPAYILVVRETHLDGTWHCHALLQCIKPVTTRDERYFDIDRYHPNIQSAKSTDKVRDYILKNPKDKWEKGTYIPRKKSFVPPGKDSSEKKPSKDEVMREIMTHATSKEEYLSLVQASLPYDWATKLSYFEYSASRLFPDIAESYTNPHPATELDLHCNETIRDWLEPNIYQNAPGTRKRSLYIVGPTRTGKTTWARSLGRHNYWQNNIDWSSYDEEALYNVVDDIPFKFCPCWKQLVGCQTDYIVNPKYGKRRKVARKSIPTIILANEDEDWLRDMTPAQQSYFEANCDQYTLLSGEKFFQTE

PanSV-G [YT-Coc-g385-2008] MSTEGSTSLNVTATGRHTIRSFRHRNVNTFLTYSKCPLEPEFIGEHLFRLSKDYEPAYILVVRETHLDGTWHCHALLQCIKPVTTRDERYFDIDRYHPNIQSAKSTDKVRDYILKNPKDKWEKGTYIPRKKSFVPPGKDSSEKKPSKDEVMREIMTHATSKEEYLSLVQASLPYDWATKLSYFEYSASRLFPDIAESYTNPHPATELDLHCNETIRDWLEPNIYQNVPGTRKRSLYIVGPTRTGKTTWARSLGRHNYWQNNIDWSSYDEEALYNVVDDIPFKFCPCWKQLVGCQTDYIVNPKYGKRRKVARKSIPTIILANEDEDWLRDMTPAQQSYFEANCDQYTLLSGEKFFRTE

PanSV-G [YT-Tsa-g386-2008] MSTEGSTSLTVTATGRHTIRSFRHRNVNTFLTYSKCPLEPEFIGEHLFRLTKDYEPAYILVVRETHLDGTWHCHALLQCIKPVTTRDERYFDIDRYHPNIQSAKSTDKVRDYILKNPKDKWEKGTYIPRKKSFVPPGKDSSEKKPSKDEVMREIMTHATSKEEYLSLVQASLPYDWATKLSYFEYSASRLFPDIAESYTNPHPATELDLHCNETIRDWLEPNIYQNAPGTRKRSLYIVGPTRTGKTTWARSLGRHNYWQNNIDWSSYDEEALYNVVDDIPFKFCPCWKQLVGCQTDYIVNPKYGKRRKVARKSIPTIILANEDEDWLRDMTPAQQSYFEANCDQYTLLSGEKFFRTE

PanSV-G [YT-Com-g383-2008] MSTEGSTSLNVTATGRHTIRSFRHRNVNTFLTYSKCPLEPEFIGEHLFRLSKDYEPAYILVVRETHLDGTWHCHALLQCIKPVTTRDERYFDIDRYHPNIQSAKSTDKVRDYILKNPKDKWEKGTYIPRKKSFVPPGKDSSEKKPSKDEVMREIMTHATSKEEYLSLVQASLPYDWATKLSYFEYSASRLFPDIAESYTNPHPPTELDLHCNETIRDWLEPNIYQNAPGTRKRSLYIVGPTRTGKTTWARSLGRHNYWQNNIDWSSYDEEALYNVVDDIPFKFCPCWKQLVGCQTDYIVNPKYGKRRKVARKSIPTIILANEDEDWLRDMTPAQQSYFEANCDQYTLLSGEKFFQTE

PanSV-D [NG-Ifo-g91-2006] MSTVGSSSE-----SRHSVRCFRHRNANTFLTYSKCPLEPEFIGEHLFRLTKDFEPAYILVVRETHTDGTWHCHALLQCIKPVTTRDERYFDIDRYHPNIQSAKSTDKVRDYILKDPKDKWEKGTYIPRKKSFVPPGKENSEKKPSKDEVMREIMTHATSKEEYLSLVQTSLPYDWATKLSYFEYSASRLFPDIAESYTNPHPATELDLHCNETIRDWLEPNIYQNAPGTRKRSLYIVGPTRTGKTTWARSLGRHNYWQNNIDWSSYDEEALYNVVDDIPFKFCPCWKQLVGCQTDYIVNPKYGKRRKVAKKSIPTIILANEDEDWLRDMTPAQLSYFEANCDQYILLSGEKFFQTE

PanSV-D [NG-Ola-g242-2007] MSTVGSSSE-----SRHSVRCFRHRNANTFLTYSKCPLEPEFIGEHLFRLTRDFEPAYILVVRETHTDGTWHCHALLQCIKPVTTRDERYFDIDRYHPNIQSAKSTDKVRDYILKDPKDKWEKGTYIPRKKSFVPPGKENSGKKPSKDEVMREIMTHATSKEEYLSLVQTSLPYDWATKLSYFEYSASRLFPDIAESYTNPHPATELDLHCNETIRDWLEPNIYQNAPGTRKRSLYIVGPTRTGKTTWARSLGRHNYWQNNIDWSSYDEEALYNVVDDIPFKFCPCWKQLVGCQTDYIVNPKYGKRRKVAKKSIPTIILANEDEDWLRDMTPAQLSYFEANCDQYILLSGEKFFQTE

PanSV-H [NG-Jic15-PNP-1997] MST----SLSVTSDGRHSVRCFRHRNANTFLTYSKCPLEPEFIGEHLFRLTKDFEPAYILVVRETHQDGTWHCHALLQCIKPVTTRDERYFDIDRYHPNIQSAKSTDKVRDYILKDPKDKWEKGTYIPRKKCFVPPGKEPAEKKPSKDEVMKEIMTHATSREEYLSLVQSSLPYDWATKLSYFEYSASRLFPDIAETFTSPHPASDPDLLCNETLQDWLEPNIYQITPGARKTSLYIVGPTRTGKTSWARSLGRHNYWQNNVDWSSYDEEALYNVVDDIPFKFCPCWKQLVGCQTDYIVNPKYGKRRKVAKKSIPTIILANEDEDWLKEMTPAQYDYFYANCEIYVMQAGEKWYTPA

PanSV-H [CF-Bai2-Car11-2008]MST----SLSVTSDGRHSVRCFRHRNANTFLTYSRCPLEPEFIGEHLFRLTRDFEPAYILVVRETHQDGTWHCHALLQCIKPVTTRDERYFDIDRYHPNIQSAKSTDKVRDYILKDPKDKWEKGTYIPRKKSFVPPGKEPAEKKPSKDEVMKEIMTHATSREEYLSLVQSSLPYDWATKLSYFEYSASRLFPDIAETFTSPHPASDPDLLCNETLQDWLEPNIYQSTPGARKRSLYIVGPTRTGKTSWARSLGRHNYWQNNIDWSSYDEEALYNVVDDIPFKFCPCWKQLVGCQTDYIVNPKYGKRRKVAKKSIPTIILANEDEDWMKDMTPAQYDYFYANCEIYVMQAGEKWYTPA

PanSV-I [KE-Nra1-g374-2008] MET----TVGSSQTGRHTVRSFRHRNVNTFLTYSKCPLEPEFIGEHLFRLTKDYDPAYILVVRETHIDGTWHCHALLQTTKPVSTSDERYFDIDRYHPNIQSAKSTDKVRAYILKDPKDKWEKGTYIPRKKSFSPPGKEPSEKKPSKDEIMKEIMTHATSREEYLSMVQSALPYDWATKLSYFEYSASRLFPDIAETYTNPHESTDIDLLCNETVQDWLEPNIYQIIPGARKRSLYIVGPTRTGKTSWARSLGRHNYWQNNIDWSSYDEEALYNVVDDIPFKFCPCWKQLVGCQKDYIVNPKYGKRRKVAKKSIPTIILANKDEDWMKDMTPAQYEYFYENCEIYVMQEGEKWYTPS

PanSV-I [KE-Nra2-g375-2008] MET----TVESSQTGRHTVRSFRHRNVNTFLTYSRCPLEPEFIGEHLLRLTKDYDPAYILVVRETHIDGTWHCHALLQTTKPVSTSDERYFDIDRYHPNIQSAKSTDKVRAYILKDPKDKWEKGTYIPRKKSFSPPGKEPSEKKPSKDEIMKEIMTHATSREEYLSMVQSALPYDWATKLSYFEYSASRLFPDIAEPYTNPHENTDIDLLCNETVQDWLEPNIYQIIPGARKRSLYIVGPTRTGKTSWARSLGRHNYWQNNIDWSSYDEEALYNVVDDIPFKFCPCWKQLVGCQKDYIVNPKYGKRRKVAKKSIPTIILANKDEDWMKDMTPAQYEYFYENCEIYVMQEGEKWYTPS

PanSV-I [KE-Jic13-PKPB-1997]MET----TVDSSQTGRHTVRSFRHRNVNTFLTYSKCPLEPEFIGEHLFRLTKDFDPAYILVVRETHIDGTWHCHALLQTTRPVSTSDERYFDIDRYHPNIQSAKSTDKVRSYILKDPKEKWEKGTYIPRKKSFSPPGKEPSEKKPSKDEVMREIMTHATSREEYLSMVQSALPYDWATKLSYFEYSASRLFPDIAEPYTNPHEATDIDLLCNETVQDWLEPNIYQALPGSRKRSLYIVGPTRTGKTSWARSLGRHNYWQNNIDWSSYDEEALYNVVDDIPFKFCPCWKQLVGCQKDYIVNPKYGKRRKVAKKSIPTIILANKDEDWMKDMTPAQYEYFYENCEIYVMQEGEKWYTPS

Rolling circle replication motifs[1]
