# Supplementary material for: Comparative analysis of Panicum streak virus and Maize streak virus diversity, recombination patterns and phylogeography
Source: Virol J. 2009 Nov 10;6:194. doi: 10.1186/1743-422X-6-194 (PMC2777162; doi:10.1186/1743-422X-6-194)
Supplement: Additional file 6 — Annotated predicted RepA amino acid sequence alignments. Annotated predicted RepA amino acid sequence alignments of 23 PanSV isolates. Potential rolling circle replication motifs and interaction domains inferred by analogy with MSV and Wheat dwarf virus are highlighted. [1] Koonin & Ilyina. 1992. J Gen Virol, 73:2763; [2] Horvath et al. 1998. Plant Mol. Biol. 38:699; [3] Xie et al. 1995. EMBO J. 14:4073; [4] Xie et al. 1999. Plant Mol. Biol. 39:647. [file 1743-422X-6-194-S6.doc]

PanSV-A [ZM-Nya-g180-2007] MST----SLSITSDGRHSVRSFRHRNANTFLTYSKCPLEPEFIGEHLFRLTKDFEPAYILVVRETHQDGTWHCHALLQCIKPVTTRDERYFDIDRYHPNIQSAKSTDKVREYILKDPKDKWEKGTYIPRKKSFVAPGK-NTEKKPSKDEVMKEIMTHATSRAEYLSLVQTSLPYDWATKLSYFEYSASRLFPDIAEPYSNPHPATDPDLLCNETLQDWLEPNIYQVSPKAYMLLEPSCLSLEQAKADLEWLSETTRLLQEQESAASTSSAQHGQVKHPGPEASDDTTTGRIISTGLHMMKKLSTMSLTTFPSSSVRAGSSSSAVKKTT

Rolling circle replication motifs[1]

Oligomerisation domain[2]

Retinoblastoma binding protein binding motif[3]

Potential transactivation domain (MSV)[2]

Potential GRAB binding protein binding domain[4]

PanSV-A [ZA-Bak-M34-2005] MST----SLSITSDGRHSVRSFRHRNANTFLTYSKCPLEPEFIGEHLFRLTKDFEPAYILVVRETHQDGTWHCHALLQCIKPVTTRDERYFDIDRYHPNIQSAKSTDKVREYILKDPKDKWEKGTYIPRKKSFVPPGKENSEKKPSKDEVMKEIMTHATSRAEYLSLVQTSLPYDWATKLSYFEYSASRLFPDIAEPYSNPHPATDPDLLCNETLQDWLEPNIYQVSPKAYMLLEPSCLSLEQAKADLEWLSETTRLLQEQESAASTSSAQHGQVKHPGPEASDGTTTGRTISTGLHMMKKLSTMSLTTFPSSSVRAGSSSSAVKRTT

PanSV-A [ZA-For-g191-2007] MST----SLSITSDGRHSVRSFRHRNANTFLTYSKCPLEPQFIGEHLFRLTKDFEPAYILVVRETHQDGTWHCHALLQCIKPVTTRDERYFDIDRYHPNIQSAKSTDKVREYILKDPKDKWEKGTYIPRKKSFVPPGKENSEKKPSKDEVMKEIMTHATSRAEYLSLVQTSLPYDWATKLSYFEYSASRLFPDIAEPYSNPHPATDPDLLCNETLQDWLEPNIYQVSPKAYMLLEPSCLSLEQAKADLEWLSETTRLLQEQESAASTSSAQHGQVKHPGPEASDDTTTGKIISTGLHMMKKLSTMSLTTFPSSSVRAGSSSSAAKKTT

PanSV-A [ZA-Kar-1994] MST----SLSITSDGRHSVRSFRHRNANTFLTYSKCPLEPEFIGEHLFRLTKDFEPAYILVVRETHQDGTWHCHALLQCIKPVTTRDERYFDIDRYHPNIQSAKSTDKVREYILKDPKDKWEKGTYIPRKKSFVPPGKENSEKKPSKDEVMKEIMTHATSRAEYLSLVQTSLPYDWATKLSYFEYSASRLFPDIAEPYSNPHPATDPDLLCNETLQDWLEPNIYQVSPKAYMLLEPSCLSLEQAKADLEWLSETTRLFQEQESEASTSSAQHGQVKHPGPEASDGTTTGRITSTGLHTMKKLRTMSWTTFPSSSVRAGSSSSAVKKTT

PanSV-A [ZA-Ill-g263-2008] MST----SLSITSDGRHSVRSFRHRNANTFLTYSKCPLEPEFIGEHLFRLTKDFEPAYILVVRETHQDGTWHCHALLQCIKPVTTRDERYFDIDRYHPNIQSAKSTDKVREYILKDPKDKWEKGTYIPRKKSFVPPGKENSEKKPSKDEVMKEIMTHATSRAEYLSLVQTSLPYDWGTKLSYFEYSASRLFPDIAEPYSNPHPATDPDLLCNETLQDWLEPNIYQVSPKAYMLLEPSCLSLEQAKADLEWLSETTRLFQEQESEASTSSAQHGQVKHPGPEALDDTTTGRIISTGLHTMKKLRTMSWTTFPSSSVRAGSSSSAVKKTT

PanSV-A [MZ-Nac1-2009] MST----SLSITSDGRHSVRSFRHRNANTFLTYSKCPLEPEFIGEHLFRLTKDFEPAYILVVRETHQDGTWHCHALLQCIKPVTTRDERYFDIDRYHPNIQSAKSTDKVREYILKDPKDKWEKGTYIPRKKSFVPPGKENSEKKPSKDEVMKEIMTHATSRAEYLSLVQTSLPYDWATKLSYFEYSASRLFPDIAEPYSNPHPATDPDLLCNETLQDWLEPNIYQVSPKAYMLLEPSCLSLEQAKADLEWLSETTRLLQEQESAASTSSAQHGQVKHPGPEASDDTTTGRIISTGLHTMKKLSTMSLTTFPSSSVRAGSSSSAVKRTT

PanSV-C [ZM-NGur-g169-2006] MST----SLSITSDGRHSVRSFRHRNANTFLTYSKCPLEPEFIGEHLFRLTKDFEPAYILVVRETHADGTWHCHALLQCIKPVTTRDERYFDIDRYHPNIQSAKSTDKVRDYILKDPKDKWEKGTYIPRKKSFSPPGKESSEKKPTKDEVMREIMTHATSREEYLSLVQSSLPYDWATKLSYFEYSASRLFPDIAETFTSPHPASDPDLLCNETLQDWLEPNIYQVSPKAYMLLEPSCLSLEQAKADLDWLAETTRLLQEQERQASTSSAQHGQVKHPGPEASDGTTTGRIISTGLHMMKKLSTMSLTTFPSSSVRAGSSSSVVRPTT

PanSV-B [KE-Ken-1991] MSTVGSSSE-----GRHSVRCFRHRNANTFLTYSKCPLEPEFIGEHLFRLTREYEPAYILVVRETHTDGTWHCHALLQCIKPCTTRDERYFDIDRYHGNIQSAKSTDKVREYILKDPKDKWEKGTYIPRKKSFVPPGKEPAEKKPTKDEVMREIMTHATSREEYLSLVQSSLPYDWATKLNYFEYSASRLFPDIAEPYTNPHPTTEYDLHCNETIEDWLKPNIYQVSPQAYKLLEPSCLSLEQAIADLEWLDDTTRMLQEKEREASTSAAQHGQVKHPGLEASDDTTTGKTISTGLHMMKKLSTMSLTTFPSSSVRAGNDSSAAKKTT

PanSV-E [KE-Jic10-PKPM-1997]MSTEGSTSLTVTPTGRHTIRSFRHRNVNTFLTYSKCPLEPEFIGEHLFRLTKDYEPAYILVVRETHTDGTWHCHALLQCIKPVTTRDERYFDIDRYHPNIQSAKSTDKVRDYILKDPKDKWEKGTYIPRKKSFSPPGKDSSEKKPSKDEVMRDIMTHATSREEYLSLVQSSLPYDWATKLSYFEYSASRLFPDIAETFTSPHPTSEPDLLCNETLQDWLEPNIYQVSPKAYMLLEPSCLSLEQAKADLDWLAETTRLLQEQERQASTSSAQHGQVKHPGLEASDDTTTGRTMSTGLHMMKKLSTMSLTTFPSSSVRAGSSSSAAKQTT

PanSV-E [KE-Nye5-g359-2008] MSTEGSTSLTVTPTGRHTIRSFRHRNVNTFLTYSKCPLEPEFIGEHLFRLTKDYEPAYILVVRETHTDGTWHCHALLQCIKPVTTRDERYFDIDRYHPNIQSAKSTDKVRDYILKDPKDKWEKGTYIPRKKSFSPPGKDSSEKKPSKDEVMREIMTHATSREEYLSLVQSSLPYDWATKLSYFEYSASRLFPDIAETFTSPHPTSDPDLLCNETLQDWLEPNIYQVSPKAYMLLEPSCLSLEQAKADLDWLAETTRLLREQERQASTSSAQHGQVKHPGLEASDDTTTGRTMSTGLHMMKKLSTMSLTTFPSSSVRAGSSSSAVKRTT

PanSV-E [KE-Nye4-g363-2008] MSTEGSTSLTVTPTGRHTIRSFRHRNVNTFLTYSKCPLEPEFIGEHLFRLTKDYEPAYILVVRETHTDGTWHCHALLQCIKPVTTRDERYFDIDRYHPNIQSAKSTDKVRDYILKDPKDKWEKGTYIPRKKSFSPPGKDSSEKKPSKDEVMREIMTHATSREEYLSLVQSSLPYDWATKLSYFEYSASRLFPDIAETFTSPHPTSDPDLLCNETLQDWLEPNIYQVSPKAYMLLEPSCLSLEQAKADLDWLAETTRLLQEQEREASTSSAQHGQVKHPGLEASDDTTTGRTMSTGLHMMKKLSTMSLTTFPSSSVRAGSSSSAVKRTT

PanSV-F [KE-Nye2-g364-2008] MSTEGSTSLTVTPTGRHTVRSFRHRNVNTFLTYSKCPLEPEFIGEHLFRLTKDYEPAYILVVRETHIDGTWHCHALLQCIKPVTTRDERYFDIDRYHPNIQSAKSTDKVREYILKDPKDKWEKGTYIPRKKSFVPPGKENSEKKPSKDEIMREIMTHATSKEEYLSLVQTSLPYDWATKLSYFEYSASRLFPDIAESYTNPHPATELDLHCNETIRDWLEPNIYQVSPKAYMLLEPSCLSLEQAKADLDWLSETTRMLQEQENAASTSSAQHGQVRQLGPEASDDTTTGRIMSTGLHMMKKLSTMSLTTFPSSSARAGSSS

PanSV-G [YT-Ben-g384-2008] MSTEGSTSLNVTATGRHTIGSFRHRNVNTFLTYSKFPLEPEFIGEHLFRLTKDYEPAYILVVRETHLDGTWHCHALLQCIKPVTTRDERYFDIDRYHPNIQSAKSTDKVRDYILKNPKDKWEKGTYIPRKKSFVPPGKDSSEKKPSKDEVMREIMTHATSKEEYLSLVQASLPYDWATKLSYFEYSASRLFPDIAESYTNPHPATELDLHCNETIRDWLEPNIYQVIPKAYMLLEPSCLSLEQAKADLEWLSETTRMLQEQENAASTSSAQHGPAKLPGPEASGDTTTGRTISTGLHTMKKLSTMSLTTFPSSSARAGSSS

PanSV-G [YT-Coc-g385-2008] MSTEGSTSLNVTATGRHTIRSFRHRNVNTFLTYSKCPLEPEFIGEHLFRLSKDYEPAYILVVRETHLDGTWHCHALLQCIKPVTTRDERYFDIDRYHPNIQSAKSTDKVRDYILKNPKDKWEKGTYIPRKKSFVPPGKDSSEKKPSKDEVMREIMTHATSKEEYLSLVQASLPYDWATKLSYFEYSASRLFPDIAESYTNPHPATELDLHCNETIRDWLEPNIYQVSPKAYMLLEPSCLSLEQAKADLEWLSETTRMFQEQENAASTSSAQHGPAKLPGPEASGDTTIGRTISTGLHMMKKLSTMSLTTFPSSSARAGSSS

PanSV-G [YT-Tsa-g386-2008] MSTEGSTSLTVTATGRHTIRSFRHRNVNTFLTYSKCPLEPEFIGEHLFRLTKDYEPAYILVVRETHLDGTWHCHALLQCIKPVTTRDERYFDIDRYHPNIQSAKSTDKVRDYILKNPKDKWEKGTYIPRKKSFVPPGKDSSEKKPSKDEVMREIMTHATSKEEYLSLVQASLPYDWATKLSYFEYSASRLFPDIAESYTNPHPATELDLHCNETIRDWLEPNIYQVSPKAYMLLEPSCLSLEQAKADLEWLSETTRMLQEQENAASTSSAQHGPAKLPGPEASGDTTTGRTISTGLHMMKKLSTMSLTTFPSSSARAGSSS

PanSV-G [YT-Com-g383-2008] MSTEGSTSLNVTATGRHTIRSFRHRNVNTFLTYSKCPLEPEFIGEHLFRLSKDYEPAYILVVRETHLDGTWHCHALLQCIKPVTTRDERYFDIDRYHPNIQSAKSTDKVRDYILKNPKDKWEKGTYIPRKKSFVPPGKDSSEKKPSKDEVMREIMTHATSKEEYLSLVQASLPYDWATKLSYFEYSASRLFPDIAESYTNPHPPTELDLHCNETIRDWLEPNIYQVSPKAYMLLEPSCLSLEQAKADLEWLSETTRMLQEQENAASTSSAQHGPAKLPGPEASGDTTTGRTISTGLHMMKKLSTMSLTTFPSSSARAGSSS

PanSV-D [NG-Ifo-g91-2006] MSTVGSSSE-----SRHSVRCFRHRNANTFLTYSKCPLEPEFIGEHLFRLTKDFEPAYILVVRETHTDGTWHCHALLQCIKPVTTRDERYFDIDRYHPNIQSAKSTDKVRDYILKDPKDKWEKGTYIPRKKSFVPPGKENSEKKPSKDEVMREIMTHATSKEEYLSLVQTSLPYDWATKLSYFEYSASRLFPDIAESYTNPHPATELDLHCNETIRDWLEPNIYQVSPKAYMLLEPSCLSLEQAKADLEWLAETTRMLQEQENAASTSSAQHGQVKQPGPEASEDTTTGKTILTGLHMMKKLSTMSLTTFPSSSVRAGSSSSAAKQTT

PanSV-D [NG-Ola-g242-2007] MSTVGSSSE-----SRHSVRCFRHRNANTFLTYSKCPLEPEFIGEHLFRLTRDFEPAYILVVRETHTDGTWHCHALLQCIKPVTTRDERYFDIDRYHPNIQSAKSTDKVRDYILKDPKDKWEKGTYIPRKKSFVPPGKENSGKKPSKDEVMREIMTHATSKEEYLSLVQTSLPYDWATKLSYFEYSASRLFPDIAESYTNPHPATELDLHCNETIRDWLEPNIYQVSPKAYMLLEPSCLSLEQAKADLEWLSETTRMLQEQENAASTSSAQHGQVKPPGPEASEDTTTGKTILTGLHMMKKLSTMSLTTFPSSSVRAGSSSSAAKQTT

PanSV-H [NG-Jic15-PNP-1997] MST----SLSVTSDGRHSVRCFRHRNANTFLTYSKCPLEPEFIGEHLFRLTKDFEPAYILVVRETHQDGTWHCHALLQCIKPVTTRDERYFDIDRYHPNIQSAKSTDKVRDYILKDPKDKWEKGTYIPRKKCFVPPGKEPAEKKPSKDEVMKEIMTHATSREEYLSLVQSSLPYDWATKLSYFEYSASRLFPDIAETFTSPHPASDPDLLCNETLQDWLEPNIYQVSPKAYMLLEPSCLSLEQAKADLDWLAETTRLLQEQERQASTSSAQHGQEKHPGPEASDATTTGKTMSTGLHMMKKLSTMSLTTFPSSSVRAGSSSSAAKQTT

PanSV-H [CF-Bai2-Car11-2008]MST----SLSVTSDGRHSVRCFRHRNANTFLTYSRCPLEPEFIGEHLFRLTRDFEPAYILVVRETHQDGTWHCHALLQCIKPVTTRDERYFDIDRYHPNIQSAKSTDKVRDYILKDPKDKWEKGTYIPRKKSFVPPGKEPAEKKPSKDEVMKEIMTHATSREEYLSLVQSSLPYDWATKLSYFEYSASRLFPDIAETFTSPHPASDPDLLCNETLQDWLEPNIYQVSPKAYMLLEPSCLSLEQAKADLHWLAETTRVLQEQEREASTSSAQHGQEKHPGPEASDATTTGRTISTGLHMMKKLSTMSLTTFPSSSVRAGSSSSAAKQTT

PanSV-I [KE-Nra1-g374-2008] METTVGSSQ----TGRHTVRSFRHRNVNTFLTYSKCPLEPEFIGEHLFRLTKDYDPAYILVVRETHIDGTWHCHALLQTTKPVSTSDERYFDIDRYHPNIQSAKSTDKVRAYILKDPKDKWEKGTYIPRKKSFSPPGKEPSEKKPSKDEIMKEIMTHATSREEYLSMVQSALPYDWATKLSYFEYSASRLFPDIAETYTNPHESTDIDLLCNETVQDWLEPNIYQVSPQAYMLLEPSCLSREQAIADLQWLSETTRLFQEQEREASTSSAQHGQVKHHGPEASDDITIGRTISTGLHTMKKLSTMSSTTFPSSSVRAGSSSSAVKKTTSSTRSTGNAAK

PanSV-I [KE-Nra2-g375-2008] METTVESSQ----TGRHTVRSFRHRNVNTFLTYSRCPLEPEFIGEHLLRLTKDYDPAYILVVRETHIDGTWHCHALLQTTKPVSTSDERYFDIDRYHPNIQSAKSTDKVRAYILKDPKDKWEKGTYIPRKKSFSPPGKEPSEKKPSKDEIMKEIMTHATSREEYLSMVQSALPYDWATKLSYFEYSASRLFPDIAEPYTNPHENTDIDLLCNETVQDWLEPNIYQVSPQAYMLLEPSCLSREQAIADLQWLSETTRLFQEQEREASTSSAQHGQVKHHGPEASDDITIGRTISTGLHMMKKLSTMSSTTFPSSSVRAGSSSSAVKKTTSSTRSTGNDAK

PanSV-I [KE-Jic13-PKPB-1997]METTVDSSQ----TGRHTVRSFRHRNVNTFLTYSKCPLEPEFIGEHLFRLTKDFDPAYILVVRETHIDGTWHCHALLQTTRPVSTSDERYFDIDRYHPNIQSAKSTDKVRSYILKDPKEKWEKGTYIPRKKSFSPPGKEPSEKKPSKDEVMREIMTHATSREEYLSMVQSALPYDWATKLSYFEYSASRLFPDIAEPYTNPHEATDIDLLCNETVQDWLEPNIYQVSPQAYMLLEPSCLSREQAIADLQWLSETTRLFQDQEREASTSSAQHGQVKHHGPEASDDTTIGRTISTGLHMMKKLSTMSSTTFPSSSVRAGSSSSAVKKTTSSTPSTGNDGR
